# Integrated analysis of doubly disadvantaged neighborhoods by considering both green space and blue space accessibility and COVID-19 infection risk

**DOI:** 10.1371/journal.pone.0273125

**Published:** 2022-11-02

**Authors:** Dong Liu, Mei-Po Kwan

**Affiliations:** 1 Human Environments Analysis Laboratory, The University of Western Ontario, Social Sciences Centre, London, Ontario, Canada; 2 Department of Geography and Environment, The University of Western Ontario, Social Sciences Centre, London, Ontario, Canada; 3 Department of Geography and Resource Management and Institute of Space and Earth Information Science, The Chinese University of Hong Kong, Shatin, Hong Kong; University of Calabria Faculty of Engineering: Universita della Calabria, ITALY

## Abstract

The ongoing COVID-19 pandemic has taken a heavy toll on the physical and mental health of the public. Nevertheless, the presence of green and blue spaces has been shown to be able to encourage physical activities and alleviate the mental distress caused by COVID-19. However, just as the impact of COVID-19 varies by geographical region and area, the distribution of green and blue spaces is also different across different neighborhoods and areas. By using Hong Kong as the study area, we determine the local neighborhoods that suffer from both high COVID-19 infection risk as well as low green and blue space accessibility. The results show that some of the poorest neighborhoods in the territory such as Sham Shui Po, Kwun Tong and Wong Tai Sin are also among the most doubly disadvantaged in terms of COVID-19 infection risk as well as green and blue space accessibility.

## 1. Introduction

The outbreak of COVID-19 has caused immense damage to public health around the world. This major pandemic has taken a great toll not only on people’s physical health through infection but also on their mental health through various COVID-19 lockdown measures. In the meantime, green space and blue space have been shown to be associated with the improvement of people’s physical and mental health.

However, due to the unequal spatial distribution of green and blue spaces as well as COVID-19 infection risks, their impacts on different neighborhoods also vary considerably. While some neighborhoods may enjoy good accessibility to green and blue spaces while suffering to a smaller extent from the COVID-19 pandemic, other neighborhoods could be doubly disadvantaged by having very low green and blue spaces accessibility and high COVID-19 infection risk. Nevertheless, little is known currently concerning the doubly disadvantaged neighborhoods in terms of green and blue spaces accessibility and COVID-19 infection risk. This research aims at filling the gap by revealing the doubly disadvantaged neighborhoods by considering both green and blue space accessibility as well as COVID-19 infection risk.

This paper is organized as follows. The second section presents the review of the relevant literature concerning the impact of green and blue spaces on public health and COVID-19. The third section introduces the data and method used in this study. The fourth section includes the results and findings from our study. While the final section ends with a discussion and conclusion from our research.

## 2. Literature review

### 2.1 Green and blue spaces and public health

Green space has often been found to be associated with the improvement of mental and physical health (Dadvand et al., 2015 [1]; Dewulf et al., 2016 [2]; Gubbels et al., 2016 [3]; Lu, 2019 [4]; Markevych et al., 2017 [5]; Song et al., 2021 [6]; Triguero-Mas et al., 2015 [7]; Wolch, Byrne and Newell, 2014) [8]. Astell-Burt and Feng (2019) [9] found that higher exposure to tree canopy is associated with a lower probability of mental distress among 46,786 participants from the cities of Sydney, Newcastle and Wollongong in Australia. Dewulf et al. (2016) [2] found in their study in Ghent, Belgium that late middle-aged adults are more physically active in green areas compared to when they are in non-green areas. Dadvand et al. (2015) [1] revealed that surrounding greenness, especially greenness at school, is associated with improved cognitive development for primary school children in Barcelona, Spain. Flouri, Midouhas and Joshi (2014) [10] uncovered that green space is associated with fewer behavioral and emotional problems for young urban children, especially those from poor families, in England.

In addition to green space, blue space has also been found to benefit health in different aspects (Ballesteros-Olza, Gracia-de-Rentería and Pérez-Zabaleta, 2020 [11]; Garrett et al., 2019 [12]; Pearson et al. 2019 [13]; White et al., 2013) [14]. Garrett et al. (2019) [12] revealed that regular visits to blue space are associated with better health among older adults in Hong Kong. Pearson et al. (2019) [13] found that Michigan residents who live closer to the Great Lakes have lower anxiety and mood disorder hospitalizations. Hooyberg et al. (2020) [15] discovered that Belgium residents living less than 5 km from the coast report better health compared to those who live further inland. Dempsey et al. (2018) [16] uncovered that exposure to coastal blue space is associated with better mental health outcomes for older adults in Ireland.

Instead of exploring the health benefits of green space and blue space separately, an increasing number of studies have examined the effects on health by considering both green space and blue space (Amoly et al., 2014 [17]; Bezold et al., 2018 [18]; Helbich et al., 2019 [19]; Tan et al., 2021) [20]. Helbich et al. (2019) [19] found that exposure to street-view green and blue spaces is associated with improved mental health for elderly residents in Beijing. Alcock et al. (2015) [21] uncovered that green and blue spaces are associated with a lower probability of psychiatric disorders in rural England. Tan et al. (2021) [20] revealed that mixed urban green and blue spaces can lead to an increased level of physical exercise in these mixed green-blue spaces in Singapore, which in turn could promote health outcomes.

Overall, the presence of green and blue spaces has been shown to exert similarly positive effects on human health by its association with increasing physical activity, mental health and reducing mental distress.

### 2.2 Green and blue spaces during COVID-19

More green and blue spaces have been shown to be associated with lower COVID-19 infection rates in recent studies. For instance, Ciupa and Suligowski (2021) [22] observed that a higher number of green-blue spaces is associated with fewer COVID-19 infections and deaths at the county level in Poland. Kan et al. (2021a) [23] found that neighborhoods in Hong Kong with clusters of local COVID-19 confirmed cases tend to have fewer green spaces. Lu et al. (2021) [24] discovered that a higher green space ratio is associated with a lower racial disparity of COVID-19 infection rate in a study covering 135 counties in the United States. These studies indicate the alleviating effect of green and blue spaces on COVID-19 infection rates.

Besides, COVID-19 has not only taken a toll on people’s physical health but also on mental health (Cullen, Gulati and Kelly, 2020 [25]; Holingue et al., 2020 [26]; Pfefferbaum and North, 2020) [27]. Meanwhile, green and blue spaces (Berdejo-Espinola et al., 2021 [28]; Poortinga et al., 2021 [29]; Pouso et al., 2021 [30]; Venter et al., 2020) [31] have been shown to help people cope with COVID-19 physically and mentally by serving as the resilience infrastructure where people can exercise, socialize and relax during the time of a global crisis.

Poortinga et al. (2021) [29] observed that perceived access to green space is associated with better health and wellbeing for residents in Wales during and after the first peak of COVID-19 in the United Kingdom. Pouso et al. (2021) [30] revealed that green and blue spaces can help alleviate the detrimental effect of COVID-19 lockdown measures on mental health and improve people’s emotions. Ciupa and Suligowski (2021) [22] even found that more area of green-blue spaces in individual counties is linked to a lower number of COVID-19 infections and deaths among 380 counties in Poland.

Despite all the health benefits of green and blue spaces, the spatial distribution of green and blue spaces has not been equal (Liu, Kwan and Kan, 2021 [32]; Sharifi et al. 2021) [33]. Given the importance of green and blue spaces in alleviating the negative impacts of COVID-19, policymakers need to allocate more resources to neighborhoods that have both low access to green and blue spaces as well as a high COVID-19 infection risk. Nevertheless, few studies have provided insights into this aspect to policymakers and left a gap in the literature that we intend to fill with this research.

## 3. Data and methodology

### 3.1 Study area

The Hong Kong Special Administrative Region (hereafter referred to as “Hong Kong”) is selected as our study area. Due to its proximity to mainland China, the territory confirmed its first COVID-19 case imported from mainland China on January 23, 2020, and became one of the few places in the world where COVID-19 was detected in its early stage.

**Fig 1:**
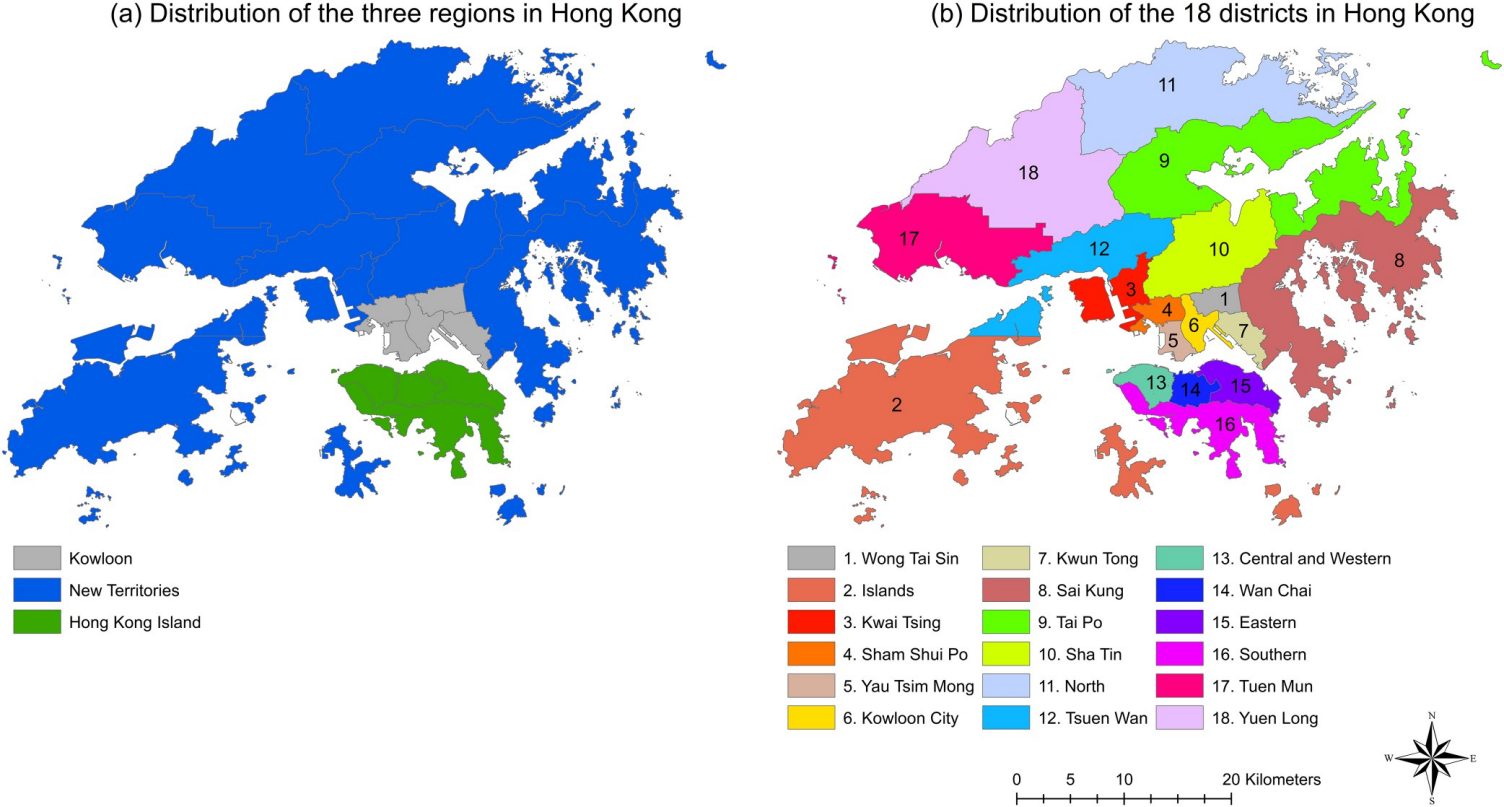
Distribution of the (a) three regions; (b) 18 districts in Hong Kong.

Hong Kong consists of three regions including Hong Kong Island, Kowloon and the New Territories. These three regions can be further divided into 18 districts, which are comprised of the districts of Central and Western, Eastern, Southern and Wan Chai in the region of Hong Kong Island; Yau Tsim Mong, Sham Shui Po, Wong Tai Sin, Kwun Tong and Kowloon City in the region of Kowloon; Islands, Yuen Long, Kwai Tsing, Tuen Mun, North, Sai Kung, Tsuen Wan, Tai Po and Sha Tin in the region of New Territories. The distributions of regions and districts in Hong Kong are presented in Fig 1.

**Fig 2:**
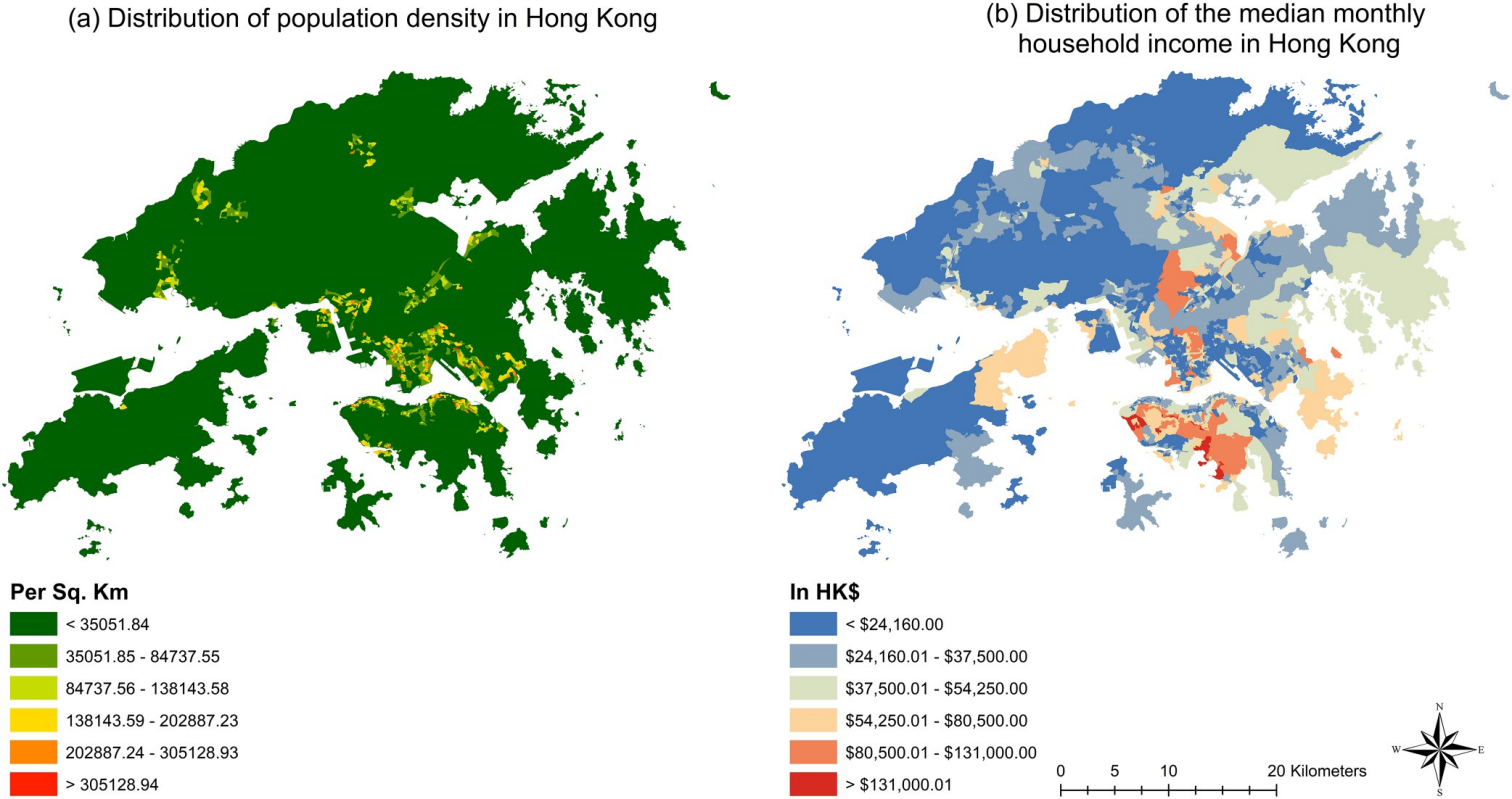
Distribution of (a) population density; (b) median monthly household income in Hong Kong.

Districts in Kowloon and Hong Kong Island are developed earlier and much more densely populated in comparison with those in the New Territories. While residents living in districts in the New Territories are mostly concentrated in a few later-developed new towns spreading across the region. In terms of economic well-being, households in districts on Hong Kong Island are generally wealthier compared to households in districts in Kowloon and the New Territories. The distributions of population density and median monthly household income are shown in Fig 2. The detailed median monthly household income at the LSBG level is provided in S1 Table in the S1 Appendix.

To better illustrate the distribution of median monthly household income by district, we proceed to produce Table 1 according to 2016 Population By-census data from the Census and Statistics Department (2017) [34].

**Table 1:** Median monthly household income (HK$) by district.

District	Median Monthly Household Income (HK$)
Sham Shui Po	$20,600
Kwun Tong	$20,800
Kwai Tsing	$21,200
North	$21,500
Wong Tai Sin	$22,000
Tuen Mun	$22,500
Yuen Long	$22,800
Yau Tsim Mong	$25,800
Tai Po	$26,200
Sha Tin	$26,800
Kowloon City	$26,900
Islands	$28,900
Tsuen Wan	$29,200
Southern	$30,700
Eastern	$31,300
Sai Kung	$31,500
Central and Western	$39,500
Wan Chai	$40,000
Hong Kong (Overall)	$25,200

As shown in Table 1, there are seven districts with lower median monthly household income than the overall median monthly household income of Hong Kong, which include Sham Shui Po, Kwun Tong, Kwai Tsing, North, Wong Tai Sin, Tuen Mun and Yuen Long. Among these seven districts, three (i.e., Sham Shui Po, Kwun Tong and Wong Tai Sin) are within the region of Kowloon; while four (i.e., Kwai Tsing, North, Tuen Mun and Yuen Long) are within the region of New Territories. The top five districts with the highest median monthly household income are comprised of Wan Chai, Central and Western, Sai Kung, Eastern and Southern. This shows that all districts of Hong Kong Island have a higher median monthly household income among all districts in Hong Kong.

### 3.2 Data source

The study unit for this research is the Large Street Block Group (LSBG). LSBG is the smallest geographic area in Hong Kong for which census data are available. Each LSBG consists of a street block with similar demographic characteristics (Kan et al., 2021b [35]; Liu et al., 2022) [36]. The study period for this research covers the first fours waves of COVID-19 by Hong Kong, which spans from January 23, 2020, to May 31, 2021.

As for the sources of data, first of all, the socioeconomic data (e.g., median monthly household income, population, population density) are obtained from the 2016 Population By-census provided by the Hong Kong Census and Statistics Department. Second, the land use (i.e., green space and blue space) data are acquired from the Planning Department. According to the Planning Department, in Hong Kong, green space includes country parks, hiking trails, open and recreation spaces, woodland, shrubland and grassland; while blue space includes rivers, streams, harbor, wetlands, marine parks/reserves, beaches, artificial lakes and reservoirs (Planning Department, 2016) [37].

Third, the road network data are provided by the Transport Department. Finally, the COVID-19 data including the locations of the confirmed cases are obtained from the Centre for Health Protection via an open-access government website.

### 3.3 Methodology

We first employ the kernel density estimation (KDE) method to measure the risk of COVID-19 infection in the study area. This study specifically uses the Epanechnikov kernel for the KDE as shown in Eq (1).

$$K_{\;}\left(d\right)=\left\{\begin{array}{ll} \frac{3}{4}\left(1-|d^{2}|\right) & \left|d\right|<1,\\ 0 & otherwise \end{array}\right.$$ (1)

where *K* represents the Epanecknikov kernel value, *d* is the ratio of distance between the center point of a kernel and a case location to bandwidth. The cell size and bandwidth are set at 100 m × 100 m and 1 km respectively. These are the parameters used in past studies on Hong Kong (e.g., Huang and Kwan, 2021) [38].

After measuring COVID-19 infection risk, we proceed to measure the accessibility to green and blue spaces together by the enhanced two-step floating catchment area (E2SFCA) method. The operationalized form of the E2SFCA method used by our study is shown in Eq (2).

$$G_{j}=\frac{M_{j}}{\sum_{o\in \left\{d_{oj}\leq T\right\}}R_{o}\times EXP\left(\frac{-d_{oj}}{\mu }\right)}$$ (2)

where *G*_*j*_ *i*s the ratio of combined green and blue area-to-population for LSBG *j*; *M*_*j*_ is the area of the combined green and blue spaces in LSBG *j*; *R*_*o*_ is the total population in LSBG *o* whose centroid is within the travel distance threshold from the centroid of LSBG *j*; *d*_*oj*_ is the distance between the centroids of LSBG *o* and LSBG *j*; *T* is the walking distance threshold whose value is set as 800 meters. The threshold value is determined based on the walking distance threshold commonly used in existing literature (Cardozo, García-Palomares and Gutiérrez, 2012 [39]; Coombes, Jones and Hillsdon, 2010) [40]. *μ* is the Gaussian distance decay function parameter.

$$A_{i}=\sum_{j\in \left\{d_{ij}\leq T\right\}}G_{j}$$ (3)

where *A*_*i*_ is the accessibility to green and blue spaces for LSBG *i*; *d*_*ij*_ is the distance between the centroids of LSBG *i* and LSBG *j*.

After obtaining the COVID-19 risk score as well as the green and blue spaces accessibility score, we calculate the medians of normalized risk score and the accessibility score. Furthermore, the LSBGs that have higher-than-median risk scores but lower-than-median accessibility scores are considered as the doubly disadvantaged LSBGs that are limited in accessing green/blue spaces and more vulnerable to enhanced mental distress during COVID-19.

## 4. Results and findings

**Fig 3:**
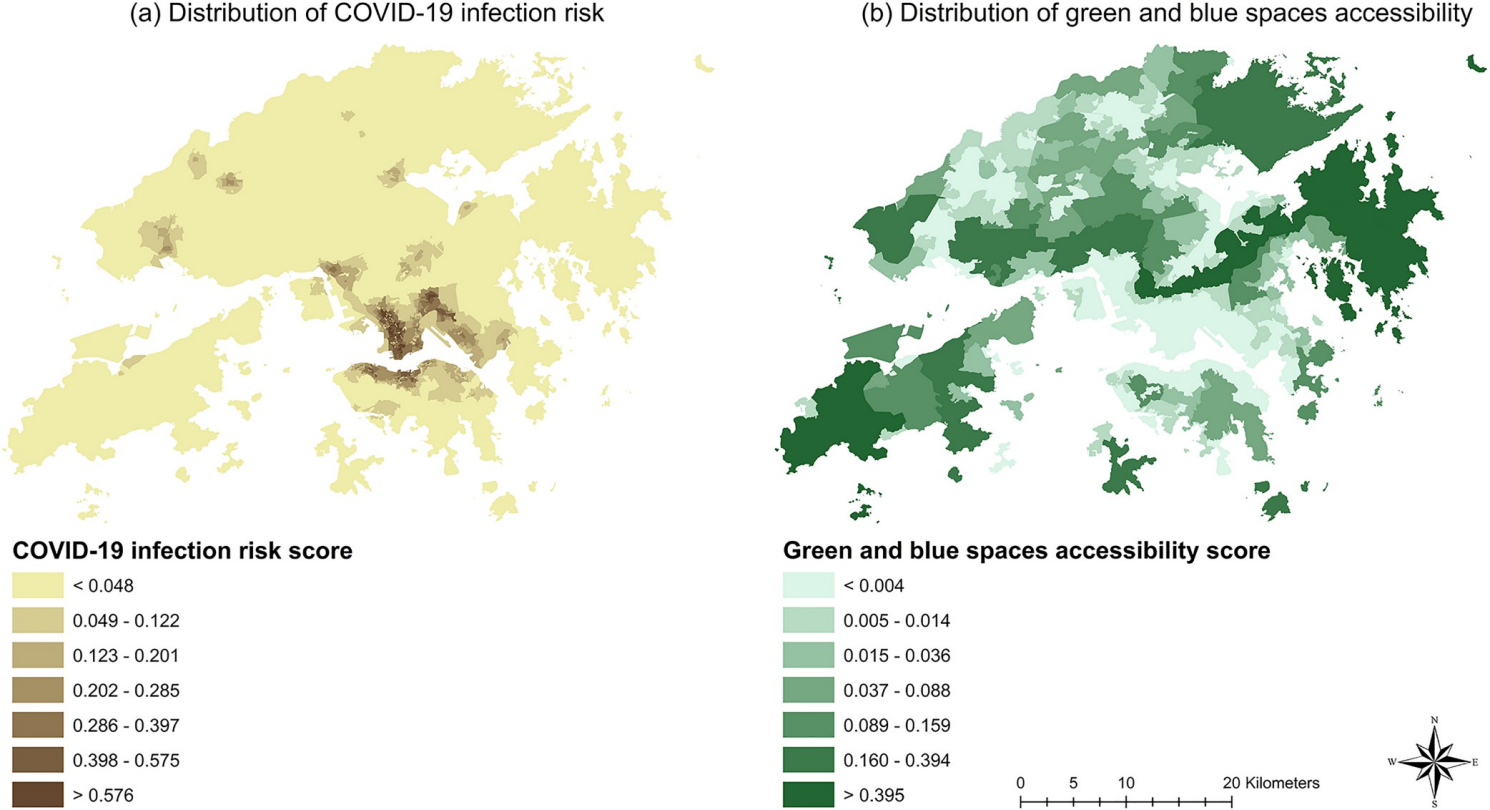
Distribution of (a) COVID-19 infection risk; (b) green and blue spaces accessibility.

**Fig 4:**
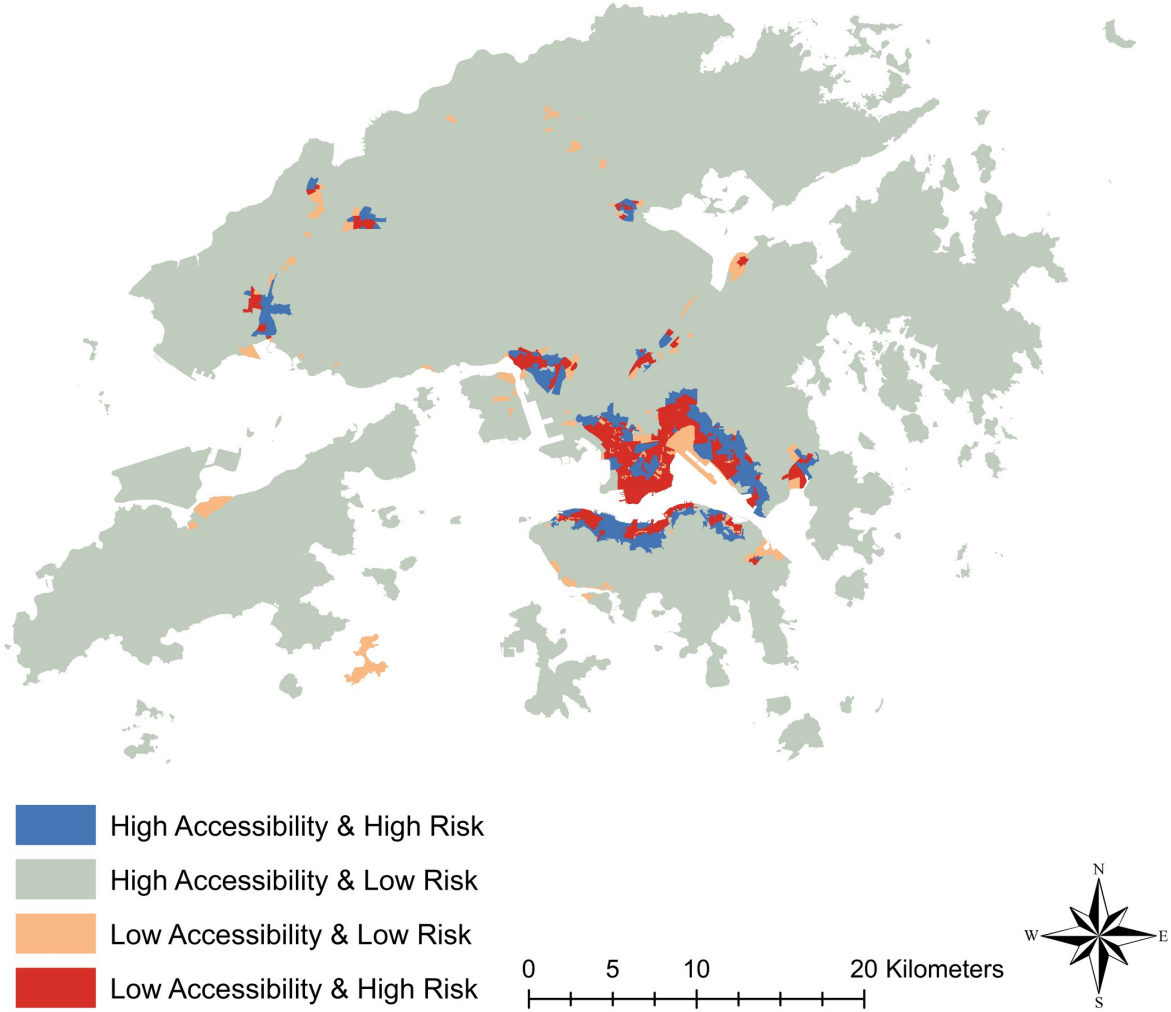
Comparison of green and blue spaces accessibility against COVID-19 infection risk.

Based on Eqs (1)–(3), we obtain the distributions of the green and blue spaces accessibility as well as COVID-19 infection risk, which can be seen in Figs 3 and 4. The results of the COVID-19 infection risk score as well as green and blue spaces accessibility scores at the LSBG level are provided in S2 Table in the S1 Appendix.

As shown by Fig 3(a), most of the LSBGs with high COVID-19 infection risk are located in the districts of Sham Shui Po, Yau Tsim Mong, Kowloon City, Wong Tai Sin and Kwun Tong in the region of Kowloon as well as the districts of Central and Western, Wan Chai and Eastern in the northern part of Hong Kong Island. As for the New Territories, it can be seen that clusters with high COVID-19 infection risk spread across the region and overlap largely with the new towns including Tsuen Wan New Town, Sha Tin New Town, Tuen Mun New Town, Yuen Long New Town, Tin Shui Wai New Town, Tai Po New Town, Fanling-Sheung Shui New Town and Tseung Kwan O New Town in the New Territories. The new towns were developed relatively later compared to the traditional population centers in Kowloon and on Hong Kong Island. The planning and development of new towns started in the 1950s in the New Territories were aimed at accommodating the influx of refugees from mainland China and relieving the shortage of land for residential development in Kowloon and on Hong Kong Island. Due to the relatively recent development of the new towns, they are heavily characterized by transit-oriented development. Moreover, due to the Country Parks Ordinance, large swathes of the land in Hong Kong, especially in the New Territories, are occupied by country parks that are designated as protected natural spaces and cannot be used for land development. Therefore, under the restriction of the Country Parks Ordinance, new towns cannot expand freely as needed as the population increases and need to spread around to accommodate more people. The combination of transit-oriented development and scarcity of legally developable land in the New Territories led to the development of high-density residential blocks surrounding major transit hubs spreading around the New Territories, which explains the high population density clusters in the New Territories as shown by Fig 2(a). Due to the high population density, LSBGs in new towns in the New Territories are showing a similar level of COVID-19 infection risk as to the densely populated LSBGs in Kowloon and on Hong Kong Island. Overall, it can be noticed that the LSBGs with high COVID-19 infection risk overlap with large parts of the LSBGs with high population density, which facilitates the spread of COVID-19 among the residents living in those densely populated LSBGs.

As for the distribution of green and blue space accessibility, we can see from Fig 3(b) that LSBGs with lower COVID-19 infection risk appear to have higher accessibility; while LSBGs with higher COVID-19 infection risk appear to have lower accessibility. This may be explained by that LSBGs with lower COVID-19 infection risk tend to also have lower population density as shown by Figs 2(a) and 3(a) and these places are usually dominated by or near large swathes of country parks which limit any major residential development in those LSBGs. Therefore, these LSBGs tend to have much better accessibility to green and blue spaces. In comparison, due to the land development restriction from the Country Park Ordinance, LSBGs with major residential land use (i.e., high population density), especially those in Kowloon and on Hong Kong Island, are usually accompanied by major commercial and industries land uses, which further limits the land available for public green and blue spaces in those LSBGs.

Although Fig 3 can provide the geographical distribution of COVID-19 infection risk as well as green and blue spaces accessibility, it is hard to tell from Fig 3 alone which districts have a higher percentage of LSBGs considered as doubly disadvantaged (i.e., high percentage of at-risk LSBGs but low percentage of LSBGs with low accessibility to blue and green spaces). Therefore, by using the median value as the benchmark value, we proceed to produce Table 2 that includes the district-level percentages of LSBGs with lower-than-median accessibility scores and higher-than-median risk scores and use such information to further obtain the percentage of LSBGs considered doubly disadvantaged in each district.

**Table 2:** Percentage of LSBGs considered disadvantaged in terms of green & blue spaces accessibility and COVID-19 infection risk.

District	% of LSBGs with lower-than-median accessibility score	% of LSBGs with higher-than-median risk score	% of LSBGs considered doubly disadvantaged
Yau Tsim Mong	92.59%	84.44%	79.26%
Wong Tai Sin	76.47%	89.71%	72.06%
Kowloon City	85.82%	74.63%	67.91%
Sham Shui Po	70.83%	76.67%	58.33%
Central & Western	56.86%	72.55%	47.06%
Kwun Tong	51.25%	90.00%	46.25%
Wan Chai	53.85%	62.82%	44.87%
Eastern	54.90%	56.21%	37.25%
Tsuen Wan	44.64%	44.64%	35.71%
Kwai Tsing	49.15%	32.20%	20.34%
Sai Kung	24.64%	24.64%	17.39%
Tai Po	22.78%	26.58%	15.19%
Sha Tin	33.33%	24.76%	14.29%
Yuen Long	22.46%	21.74%	13.77%
Tuen Mun	22.22%	30.86%	7.41%
Islands	31.03%	0.00%	0.00%
North	16.46%	0.00%	0.00%
Southern	24.56%	0.00%	0.00%

As shown in Table 2, among all 18 districts, the top five districts by the percentage of doubly disadvantaged LSBGs include Yau Tsim Mong (79.26%), Wong Tai Sin (72.06%), Kowloon City (67.91%), Sham Shui Po (58.33%) as well as Central and Western (47.06%). Out of these five districts with the highest percentage of doubly disadvantaged LSBGs, four of them lie in the Kowloon region. The only district in the Kowloon region that is not in the top five is Kwun Tong, which ranks sixth by the percentage of doubly disadvantaged LSBGs.

Meanwhile, the bottom five districts by the percentage of doubly disadvantaged LSBGs include Islands (0.00%), North (0.00%), Southern (0.00%), Tuen Mun (7.41%) and Yuen Long (13.77%). Out of the five districts with the lowest percentage of doubly disadvantaged LSBGs, four of them lie in the New Territories region. In addition, among the bottom ten districts by the percentage of doubly disadvantaged LSBGs, all, except the Southern district, lie in the New Territories.

Overall, Fig 3 and Table 2 appear to show that many of the doubly disadvantaged LSBGs are concentrated in the densely populated low-income districts. To confirm whether population density and income are associated with LSBGs being doubly disadvantaged, we conduct a multinomial logistic regression analysis by using population density and median monthly household income as the independent variables to predict whether an LSBG is doubly disadvantaged. The results are shown in Table 3.

**Table 3:** Multinomial logistic regression analysis of the doubly disadvantaged LSBGs.

	B	Std. Error	Wald	df	Sig.	Exp(B)	95% Confidence Interval for Exp(B)	
							Lower Bound	Upper Bound
Intercept	0.70143	0.1396	25.2362	1	5.07E-07			
Median Monthly Household Income	0.00002	0.0000	26.8700	1	2.17608E-07 ***	1.0000	1.0000	1.0000
Population Density	-0.00691	0.0007	105.8900	1	7.79434E-25 ***	0.9931	0.9918	0.9944

* P ≤ 0.05.

** P ≤ 0.01.

*** P ≤ 0.001.

In the multinomial logistic regression analysis, the reference category is set as the doubly disadvantaged LSBGs. As shown in Table 3, both median monthly household income and population density are statistically significant variables in determining the likelihood of an LSBG being doubly disadvantaged. Specifically, the higher the median monthly household income, the lower the likelihood an LSBG is doubly disadvantaged; while the higher the population density, the higher the likelihood the LSBG is doubly disadvantaged. Therefore, high population density and low median monthly household income are associated with a higher likelihood for an LSBG being doubly disadvantaged.

In summary, it can be seen that districts in the Kowloon region face the most serious issue and districts in the New Territories region suffer the least serious issue in terms of doubly disadvantaged LSBGs with the districts on Hong Kong Island lying in between.

## 5. Discussion and conclusion

In this study, we analyze the doubly disadvantaged neighborhoods at the LSBG level in Hong Kong by taking into consideration both green and blue space accessibility as well as COVID-19 infection risk. The green and blue spaces have been traditionally shown to be associated with a wide range of physical and mental health benefits including the facilitation of socially distanced outdoor exercises, reduction of anxieties and mood disorders as well as promotion of relaxed feelings and those benefits have become even more significant in light of the lockdown and restriction measures imposed by the authorities in light of the COVID-19 pandemic. Given the significance of green and blue spaces for public health especially during COVID-19, the results from our study provide policymakers with timely reference in regards to the disadvantaged neighborhoods having not only excessive exposure to COVID-19 infection risk but also low accessibility to green and blue spaces, which can inform the formulation of intervention measures by the authorities aimed at helping those doubly disadvantaged neighborhoods safely go through this immense public health challenge.

Overall, we found that all five districts within Kowloon, which include Yau Tsim Mong, Sham Shui Po, Wong Tai Sin, Kwun Tong and Kowloon City, have the highest percentage of doubly disadvantaged LSBGs with high COVID-19 infection risk and low accessibility to green and blue spaces. Many of the districts with a high percentage of doubly disadvantaged LSBGs in Kowloon such as Sham Shui Po, Wong Tai Sin and Kwun Tong also rank in the bottom in terms of median monthly household income as shown by Table 1. This result aligns with previous findings that low-income neighborhoods tend to have lower access to green and blue spaces (Astell-Burt and Feng, 2021 [9]; Liu, Kwan and Kan, 2021) [32] and higher COVID-19 infection rates (Hong et al., 2021 [41]; Yancy, 2020) [42]. Our study further confirms the findings from other studies that poor neighborhoods, with less access to green and blue spaces as well as higher infection risks, tend to be more vulnerable during the COVID-19 pandemic. The reason that poor neighborhoods are found to be disadvantaged in accessing green and blue spaces and being infected by COVID19 can be explained by the fact that these neighborhoods are usually densely populated and composed of high-density housing. As a result, there is little room for green and blue spaces in the proximity of these poor neighborhoods. On the other hand, densities in terms of population and housing also facilitate the spread of COVID-19 (Alam, 2021 [43]; DiMaggio et al., 2020 [44]; Dlamini et al., 2020) [45], which in turn drives up infection risk.

Given the fact that Kowloon has the smallest land area among all three regions as well as a long history of residential and commercial development, there has been very limited land for green and blue spaces development. Given the limited space on the surface in Kowloon, the policymakers in Hong Kong should consider diverting more resources towards the development of vertical green and blue spaces in the districts within Kowloon. Specifically, the government may consider developing green/blue roofs and facades for residential buildings especially those in the doubly disadvantaged LSBGs, which can help expand the green and blue spaces vertically. Such practice has been widely adopted in many densely populated urban areas with limited land space such as Singapore (He et al., 2021) [46]. Moreover, vertical green spaces such as those on rooftops have been shown to have similar health benefits to the green spaces on the ground (Triguero-Mas et al., 2020 [47]; Williams et al., 2019) [48]. Although the building of green and blue infrastructure is not a solution in the short-term, such infrastructure can still serve its purpose in the face of further pandemics or COVID-19 waves in the future.

Moreover, some COVID-19 lockdown measures such as the closure of public parks, trails and beaches should be re-visited as these outdoor public spaces have been shown to be associated with the improvement of people’s mental health with a very low possibility of spreading COVID-19 (Qian et al., 2021) [49]. Moreover, the closure of public green and blue spaces could further restrict socially distanced outdoor physical activity, increase mental stress and even drive people to exercise in places not suitable for social distancing such as sidewalks (Freeman and Eykelbosh, 2020) [50]. Given that the sidewalks in Hong Kong are usually narrow and packed with pedestrians, people forced to do exercises on sidewalks such as running could put themselves and other pedestrians at higher risk since vigorous physical activities are usually accompanied by intense exhalation, which could produce droplet sprays of high-velocity causing a larger amount of virus-laden particles such as SARS-CoV-2 in the environment (Suminski et al., 2022) [51]. Given the high-population density and compact nature of most residential areas in Hong Kong, most residents rely on public green and blue spaces for exercise and leisure activities. The closure of these public spaces could contribute to the detrimental development of the physical and mental health of the residents during COVID-19. Therefore, the government should be extraordinarily careful when it comes to determining the closure of those public spaces.

Besides the expansion of green and blue spaces in the doubly disadvantaged LSBGs, policymakers should also take preemptive measures, which could include frequent deep cleaning (Amankwah-Amoah et al., 2021) [52], enhanced ventilation systems (Blocken et al., 2021) [53], and regular body temperature screening with validated devices (Lippi et al., 2021) [54], for buildings in the doubly disadvantaged LSBGs. Given that dwellings in Hong Kong are mostly in high-rise buildings and the risk of vertical COVID-19 transmission risk through the drainage system in high-rise buildings (Kang et al., 2020) [55], the current restriction-testing measures taken by the Hong Kong Government for buildings with vertical COVID-19 transmission is inadequate. In addition, the government should consider taking additional measures to help inspect and renovate the drainage systems in those buildings, which tend to be old and prone to enhanced vertical transmission of COVID-19 as well as other diseases that may spread through aerosol transmission.

Furthermore, since many districts with a very high percentage of doubly disadvantaged LSBGs also rank at the bottom in terms of median monthly household income (e.g., Sham Shui Po, Kwun Tong and Wong Tai Sin) as shown in Fig 2(b), the government should consider distributing free face masks, hand sanitizers, thermometers and rapid antigen test kits to residents living in those districts in order to alleviate the economic burden related to purchasing those protective items against COVID-19.

Due to data availability, we cannot integrate the usage of different types of green and blue spaces into our analysis. Therefore, future studies may consider assigning weights to different types of green spaces based on their usage by residents as certain types of green and blue spaces could be more popular than others among the public (Schipperijn et al., 2010 [56]; Zhang et al., 2013) [57]. Moreover, the extent to which mental distress will be relieved can also vary among different types of green and blue spaces, which could also be considered by future studies. Further, also due to data availability, this research could not provide evidence on causal relationships. It mainly examines associations at the neighborhood level and thus may suffer from the issue of ecological fallacy and bias resulting from treating individuals living in the same neighborhood as socio-economically similar. Finally, like any studies using aggregate data, our study may also be affected by the modifiable areal unit problem.

## Supporting information

S1 Appendix (DOCX)
