# Supplementary material for: Integrated analysis of doubly disadvantaged neighborhoods by considering both green space and blue space accessibility and COVID-19 infection risk
Source: PLoS One. 2022 Nov 2;17(11):e0273125. doi: 10.1371/journal.pone.0273125 (PMC9629640; doi:10.1371/journal.pone.0273125)
Supplement: S1 Appendix — (DOCX) [file pone.0273125.s001.docx]

S1 Table. Median monthly household income (HK$) by LSBG

| **District** | **LSBG Object ID** | **Median Monthly Household Income** |
| --- | --- | --- |
| CENTRAL & WESTERN | 1 | $50,000 |
|  | 2 | $70,210 |
|  | 3 | $45,490 |
|  | 4 | $24,290 |
|  | 5 | $28,000 |
|  | 6 | $30,000 |
|  | 7 | $37,190 |
|  | 8 | $31,990 |
|  | 9 | $22,490 |
|  | 10 | $59,160 |
|  | 11 | $47,000 |
|  | 12 | $20,000 |
|  | 13 | $25,800 |
|  | 14 | $21,150 |
|  | 15 | $21,250 |
|  | 16 | $21,020 |
|  | 17 | $42,000 |
|  | 18 | $40,000 |
|  | 19 | $47,960 |
|  | 20 | $52,520 |
|  | 21 | $100,000 |
|  | 22 | $20,000 |
|  | 23 | $27,000 |
|  | 24 | $21,250 |
|  | 25 | $25,240 |
|  | 26 | $22,780 |
|  | 27 | $23,580 |
|  | 28 | $18,000 |
|  | 29 | $20,400 |
|  | 30 | $24,550 |
|  | 31 | $25,550 |
|  | 32 | $23,560 |
|  | 33 | $24,230 |
|  | 34 | $32,000 |
|  | 35 | $31,660 |
|  | 36 | $45,000 |
|  | 37 | $21,500 |
|  | 38 | $31,250 |
|  | 39 | $28,000 |
|  | 40 | $29,500 |
|  | 41 | $20,000 |
|  | 42 | $35,540 |
|  | 43 | $28,750 |
|  | 44 | $33,780 |
|  | 45 | $45,750 |
|  | 46 | $35,000 |
|  | 47 | $25,000 |
|  | 48 | $23,000 |
|  | 49 | $33,000 |
|  | 50 | $25,000 |
|  | 51 | $26,630 |
|  | 52 | $23,000 |
|  | 53 | $32,090 |
|  | 54 |  |
|  | 55 | $30,380 |
|  | 56 | $30,000 |
|  | 57 | $45,000 |
|  | 58 | $37,000 |
|  | 59 | $31,100 |
|  | 60 | $27,200 |
|  | 61 | $35,250 |
|  | 62 | $36,250 |
|  | 63 | $65,000 |
|  | 64 | $30,000 |
|  | 65 | $33,650 |
|  | 66 | $19,940 |
|  | 67 | $28,300 |
|  | 68 | $22,000 |
|  | 69 | $22,100 |
|  | 70 | $31,000 |
|  | 71 | $36,410 |
|  | 72 | $50,000 |
|  | 73 | $47,290 |
|  | 74 | $93,510 |
|  | 75 | $61,000 |
|  | 76 | $85,500 |
|  | 77 | $75,500 |
|  | 78 | $76,280 |
|  | 79 | $105,210 |
|  | 80 | $90,000 |
|  | 81 | $76,500 |
|  | 82 | $80,000 |
|  | 83 | $55,000 |
|  | 84 | $105,000 |
|  | 85 | $89,300 |
|  | 86 | $100,000 |
|  | 87 | $50,000 |
|  | 88 | $53,000 |
|  | 89 | $85,000 |
|  | 90 | $84,160 |
|  | 91 | $131,000 |
|  | 92 | $129,160 |
|  | 93 | $122,100 |
|  | 94 | $165,000 |
|  | 95 | $39,800 |
|  | 96 | $115,000 |
|  | 97 | $78,310 |
|  | 98 | $96,000 |
|  | 99 | $160,000 |
|  | 100 | $80,440 |
|  | 101 | $129,160 |
|  | 102 | $204,500 |
| EASTERN | 103 | $30,120 |
|  | 104 | $17,490 |
|  | 105 | $41,960 |
|  | 106 | $50,500 |
|  | 107 | $32,000 |
|  | 108 | $41,650 |
|  | 109 | $32,890 |
|  | 110 | $23,000 |
|  | 111 | $37,500 |
|  | 112 | $29,000 |
|  | 113 | $18,420 |
|  | 114 | $28,800 |
|  | 115 | $51,000 |
|  | 116 | $59,160 |
|  | 117 | $19,250 |
|  | 118 | $23,660 |
|  | 119 | $26,790 |
|  | 120 | $35,350 |
|  | 121 | $46,250 |
|  | 122 | $60,000 |
|  | 123 | $58,780 |
|  | 124 | $72,000 |
|  | 125 | $72,620 |
|  | 126 | $106,150 |
|  | 127 | $68,600 |
|  | 128 | $90,160 |
|  | 129 | $99,890 |
|  | 130 | $25,000 |
|  | 131 | $23,000 |
|  | 132 | $27,490 |
|  | 133 | $17,720 |
|  | 134 | $33,960 |
|  | 135 | $19,800 |
|  | 136 | $26,330 |
|  | 137 | $21,070 |
|  | 138 | $18,660 |
|  | 139 | $26,250 |
|  | 140 | $26,780 |
|  | 141 | $30,000 |
|  | 142 | $24,500 |
|  | 143 | $59,000 |
|  | 144 | $30,000 |
|  | 145 | $32,990 |
|  | 146 | $34,120 |
|  | 147 | $40,500 |
|  | 148 | $21,490 |
|  | 149 | $22,170 |
|  | 150 | $13,810 |
|  | 151 | $49,000 |
|  | 152 | $34,910 |
|  | 153 | $50,000 |
|  | 154 | $36,000 |
|  | 155 | $31,000 |
|  | 156 | $39,250 |
|  | 157 | $17,500 |
|  | 158 | $47,040 |
|  | 159 | $44,870 |
|  | 160 | $16,290 |
|  | 161 | $26,650 |
|  | 162 | $25,000 |
|  | 163 | $25,000 |
|  | 164 | $30,820 |
|  | 165 | $20,700 |
|  | 166 | $30,750 |
|  | 167 | $19,120 |
|  | 168 | $52,990 |
|  | 169 | $14,000 |
|  | 170 | $33,750 |
|  | 171 | $26,990 |
|  | 172 | $24,000 |
|  | 173 | $48,300 |
|  | 174 | $23,310 |
|  | 175 | $40,000 |
|  | 176 | $65,000 |
|  | 177 | $33,000 |
|  | 178 | $20,580 |
|  | 179 | $50,840 |
|  | 180 | $59,000 |
|  | 181 | $57,950 |
|  | 182 | $81,700 |
|  | 183 | $47,210 |
|  | 184 | $14,250 |
|  | 185 | $15,290 |
|  | 186 | $77,900 |
|  | 187 | $70,000 |
|  | 188 | $57,000 |
|  | 189 | $24,000 |
|  | 190 | $55,000 |
|  | 191 | $43,500 |
|  | 192 | $47,750 |
|  | 193 | $63,000 |
|  | 194 | $64,210 |
|  | 195 | $56,240 |
|  | 196 | $54,000 |
|  | 197 | $44,160 |
|  | 198 | $77,000 |
|  | 199 | $16,570 |
|  | 200 | $45,000 |
|  | 201 | $22,000 |
|  | 202 | $19,800 |
|  | 203 | $23,780 |
|  | 204 | $25,000 |
|  | 205 | $38,060 |
|  | 206 | $15,750 |
|  | 207 | $21,290 |
|  | 208 | $25,000 |
|  | 209 | $32,100 |
|  | 210 | $20,700 |
|  | 211 | $24,250 |
|  | 212 | $25,000 |
|  | 213 | $32,000 |
|  | 214 | $34,750 |
|  | 215 | $34,250 |
|  | 216 | $22,240 |
|  | 217 | $24,500 |
|  | 218 | $17,960 |
|  | 219 | $33,000 |
|  | 220 | $70,000 |
|  | 221 | $35,000 |
|  | 222 | $18,800 |
|  | 223 | $30,000 |
|  | 224 | $33,000 |
|  | 225 | $52,660 |
|  | 226 | $43,400 |
|  | 227 | $20,500 |
|  | 228 | $33,740 |
|  | 229 | $39,700 |
|  | 230 | $43,000 |
|  | 231 | $51,040 |
|  | 232 | $14,300 |
|  | 233 | $24,740 |
|  | 234 | $27,600 |
|  | 235 | $20,540 |
|  | 236 | $23,770 |
|  | 237 | $21,800 |
|  | 238 | $18,500 |
|  | 239 | $30,750 |
|  | 240 | $24,000 |
|  | 241 | $14,000 |
|  | 242 | $24,000 |
|  | 243 | $17,500 |
|  | 244 | $23,990 |
|  | 245 | $30,000 |
|  | 246 | $26,190 |
|  | 247 | $21,000 |
|  | 248 | $19,900 |
|  | 249 | $64,000 |
|  | 250 | $37,160 |
|  | 251 | $30,000 |
|  | 252 | $31,000 |
|  | 253 | $22,650 |
|  | 254 | $24,000 |
|  | 255 | $25,000 |
| ISLANDS | 256 | $25,200 |
|  | 257 | $21,800 |
|  | 258 | $23,500 |
|  | 259 | $27,000 |
|  | 260 | $18,100 |
|  | 261 | $20,000 |
|  | 262 | $21,500 |
|  | 263 | $14,160 |
|  | 264 | $23,250 |
|  | 265 | $20,730 |
|  | 266 | $23,700 |
|  | 267 | $28,100 |
|  | 268 | $24,000 |
|  | 269 | $23,750 |
|  | 270 | $22,750 |
|  | 271 | $10,000 |
|  | 272 | $12,600 |
|  | 273 | $20,640 |
|  | 274 | $20,500 |
|  | 275 | $19,000 |
|  | 276 | $38,750 |
|  | 277 | $20,800 |
|  | 278 | $19,000 |
|  | 279 | $20,330 |
|  | 280 | $57,160 |
|  | 281 | $57,000 |
|  | 282 | $18,380 |
|  | 283 | $16,000 |
|  | 284 | $11,000 |
| KOWLOON CITY | 285 | $51,000 |
|  | 286 | $62,210 |
|  | 287 | $43,250 |
|  | 288 | $49,900 |
|  | 289 | $69,210 |
|  | 290 | $54,160 |
|  | 291 | $70,450 |
|  | 292 | $84,160 |
|  | 293 | $55,000 |
|  | 294 | $114,910 |
|  | 295 | $87,000 |
|  | 296 | $51,000 |
|  | 297 | $63,990 |
|  | 298 | $52,210 |
|  | 299 | $52,500 |
|  | 300 | $35,000 |
|  | 301 | $9,920 |
|  | 302 | $28,960 |
|  | 303 | $35,000 |
|  | 304 | $30,000 |
|  | 305 | $30,000 |
|  | 306 | $45,250 |
|  | 307 | $65,000 |
|  | 308 | $87,160 |
|  | 309 | $19,280 |
|  | 310 | $19,870 |
|  | 311 | $23,660 |
|  | 312 | $31,250 |
|  | 313 | $54,000 |
|  | 314 | $19,000 |
|  | 315 | $17,500 |
|  | 316 | $30,000 |
|  | 317 | $17,460 |
|  | 318 | $25,000 |
|  | 319 | $31,850 |
|  | 320 | $26,560 |
|  | 321 | $60,000 |
|  | 322 | $24,500 |
|  | 323 | $31,500 |
|  | 324 | $29,000 |
|  | 325 | $20,000 |
|  | 326 | $20,000 |
|  | 327 | $34,180 |
|  | 328 | $23,750 |
|  | 329 | $19,040 |
|  | 330 | $25,000 |
|  | 331 | $19,000 |
|  | 332 | $20,500 |
|  | 333 | $21,250 |
|  | 334 | $49,010 |
|  | 335 | $18,520 |
|  | 336 | $24,430 |
|  | 337 | $16,800 |
|  | 338 | $18,520 |
|  | 339 | $18,000 |
|  | 340 | $26,500 |
|  | 341 | $20,000 |
|  | 342 | $23,920 |
|  | 343 | $18,930 |
|  | 344 | $15,750 |
|  | 345 | $17,770 |
|  | 346 | $30,000 |
|  | 347 | $18,110 |
|  | 348 | $27,790 |
|  | 349 | $28,830 |
|  | 350 | $20,000 |
|  | 351 | $28,750 |
|  | 352 | $19,650 |
|  | 353 | $30,040 |
|  | 354 | $21,200 |
|  | 355 | $20,800 |
|  | 356 | $18,000 |
|  | 357 | $21,250 |
|  | 358 | $15,000 |
|  | 359 | $16,740 |
|  | 360 | $17,000 |
|  | 361 | $25,000 |
|  | 362 | $19,830 |
|  | 363 | $17,000 |
|  | 364 | $21,570 |
|  | 365 | $60,000 |
|  | 366 | $25,250 |
|  | 367 | $32,000 |
|  | 368 | $25,250 |
|  | 369 | $13,500 |
|  | 370 | $19,800 |
|  | 371 | $37,000 |
|  | 372 | $29,000 |
|  | 373 | $34,340 |
|  | 374 | $50,000 |
|  | 375 | $42,000 |
|  | 376 | $33,290 |
|  | 377 | $50,160 |
|  | 378 | $50,000 |
|  | 379 | $49,750 |
|  | 380 | $59,200 |
|  | 381 | $64,160 |
|  | 382 | $17,290 |
|  | 383 | $62,750 |
|  | 384 | $15,320 |
|  | 385 | $68,960 |
|  | 386 | $14,930 |
|  | 387 | $63,000 |
|  | 388 | $60,400 |
|  | 389 | $63,200 |
|  | 390 | $41,750 |
|  | 391 | $47,000 |
|  | 392 | $14,490 |
|  | 393 | $105,320 |
|  | 394 | $78,160 |
|  | 395 | $97,740 |
|  | 396 | $88,370 |
|  | 397 | $105,320 |
|  | 398 | $82,000 |
|  | 399 | $59,290 |
|  | 400 | $116,400 |
|  | 401 | $55,000 |
|  | 402 | $49,000 |
|  | 403 | $58,760 |
|  | 404 | $97,740 |
|  | 405 | $94,500 |
|  | 406 | $94,500 |
|  | 407 | $70,000 |
|  | 408 | $90,960 |
|  | 409 | $27,000 |
|  | 410 | $24,230 |
|  | 411 | $22,210 |
|  | 412 | $17,000 |
|  | 413 | $22,230 |
|  | 414 | $23,160 |
|  | 415 | $22,230 |
|  | 416 | $19,010 |
|  | 417 | $23,560 |
|  | 418 | $14,000 |
| KWAI TSING | 419 | $50,000 |
|  | 420 | $14,000 |
|  | 421 | $20,270 |
|  | 422 | $16,850 |
|  | 423 | $17,800 |
|  | 424 | $17,800 |
|  | 425 | $18,000 |
|  | 426 | $32,580 |
|  | 427 | $22,320 |
|  | 428 | $17,300 |
|  | 429 | $38,360 |
|  | 430 | $19,490 |
|  | 431 | $18,000 |
|  | 432 | $32,870 |
|  | 433 | $16,990 |
|  | 434 | $36,000 |
|  | 435 | $22,500 |
|  | 436 | $21,130 |
|  | 437 | $18,800 |
|  | 438 | $21,790 |
|  | 439 | $40,750 |
|  | 440 | $18,850 |
|  | 441 | $17,790 |
|  | 442 | $20,000 |
|  | 443 | $28,240 |
|  | 444 | $20,150 |
|  | 445 | $55,600 |
|  | 446 | $42,410 |
|  | 447 | $18,080 |
|  | 448 | $51,500 |
|  | 449 | $21,000 |
|  | 450 | $15,000 |
|  | 451 | $29,000 |
|  | 452 | $25,000 |
|  | 453 | $33,050 |
|  | 454 | $21,900 |
|  | 455 | $25,420 |
|  | 456 | $36,250 |
|  | 457 | $40,000 |
|  | 458 | $35,000 |
|  | 459 | $33,000 |
|  | 460 | $35,000 |
|  | 461 | $15,000 |
|  | 462 | $23,000 |
|  | 463 | $25,390 |
|  | 464 | $22,650 |
|  | 465 | $37,250 |
|  | 466 | $27,200 |
|  | 467 | $42,800 |
|  | 468 | $70,700 |
|  | 469 | $31,470 |
|  | 470 | $67,050 |
|  | 471 | $25,880 |
|  | 472 | $25,500 |
|  | 473 | $29,550 |
|  | 474 | $23,740 |
|  | 475 | $14,490 |
|  | 476 | $20,920 |
|  | 477 | $60,000 |
| KWUN TONG | 478 | $13,050 |
|  | 479 | $31,000 |
|  | 480 | $27,910 |
|  | 481 | $50,000 |
|  | 482 | $65,660 |
|  | 483 | $60,000 |
|  | 484 | $60,000 |
|  | 485 | $16,500 |
|  | 486 | $63,740 |
|  | 487 | $18,690 |
|  | 488 | $15,390 |
|  | 489 | $12,550 |
|  | 490 | $30,000 |
|  | 491 | $15,500 |
|  | 492 | $12,000 |
|  | 493 | $17,500 |
|  | 494 | $15,400 |
|  | 495 | $15,500 |
|  | 496 | $15,000 |
|  | 497 | $35,000 |
|  | 498 | $47,690 |
|  | 499 | $27,500 |
|  | 500 | $21,050 |
|  | 501 | $17,000 |
|  | 502 | $23,750 |
|  | 503 | $35,000 |
|  | 504 | $25,500 |
|  | 505 | $37,000 |
|  | 506 | $15,370 |
|  | 507 | $20,370 |
|  | 508 | $15,000 |
|  | 509 | $33,800 |
|  | 510 | $22,360 |
|  | 511 | $16,100 |
|  | 512 | $16,490 |
|  | 513 | $12,750 |
|  | 514 | $20,650 |
|  | 515 | $15,000 |
|  | 516 | $21,250 |
|  | 517 | $15,900 |
|  | 518 | $17,100 |
|  | 519 | $15,300 |
|  | 520 | $16,330 |
|  | 521 | $28,500 |
|  | 522 | $16,380 |
|  | 523 | $15,300 |
|  | 524 | $15,300 |
|  | 525 | $29,650 |
|  | 526 | $27,550 |
|  | 527 | $15,000 |
|  | 528 | $11,980 |
|  | 529 | $29,000 |
|  | 530 | $14,000 |
|  | 531 | $27,700 |
|  | 532 | $33,260 |
|  | 533 | $9,230 |
|  | 534 | $50,420 |
|  | 535 | $27,000 |
|  | 536 | $38,040 |
|  | 537 | $37,000 |
|  | 538 | $19,980 |
|  | 539 | $19,920 |
|  | 540 | $15,020 |
|  | 541 | $12,000 |
|  | 542 | $56,500 |
|  | 543 | $31,000 |
|  | 544 | $17,900 |
|  | 545 | $22,490 |
|  | 546 | $26,000 |
|  | 547 | $39,300 |
|  | 548 | $40,000 |
|  | 549 | $16,320 |
|  | 550 | $29,000 |
|  | 551 | $20,510 |
|  | 552 | $27,000 |
|  | 553 | $60,000 |
|  | 554 | $32,400 |
|  | 555 | $24,500 |
|  | 556 | $32,000 |
|  | 557 | $27,400 |
| NORTH | 558 | $19,200 |
|  | 559 | $23,750 |
|  | 560 | $29,990 |
|  | 561 | $15,490 |
|  | 562 | $15,480 |
|  | 563 | $23,000 |
|  | 564 | $19,500 |
|  | 565 | $24,800 |
|  | 566 | $26,940 |
|  | 567 | $15,500 |
|  | 568 | $29,610 |
|  | 569 | $36,750 |
|  | 570 | $20,000 |
|  | 571 | $16,920 |
|  | 572 | $19,120 |
|  | 573 | $19,120 |
|  | 574 | $17,650 |
|  | 575 | $28,000 |
|  | 576 | $27,000 |
|  | 577 | $25,000 |
|  | 578 | $26,000 |
|  | 579 | $21,000 |
|  | 580 | $20,850 |
|  | 581 | $28,860 |
|  | 582 | $35,750 |
|  | 583 | $57,000 |
|  | 584 | $14,380 |
|  | 585 | $19,870 |
|  | 586 | $27,790 |
|  | 587 | $31,000 |
|  | 588 | $18,500 |
|  | 589 | $17,980 |
|  | 590 | $23,750 |
|  | 591 | $15,340 |
|  | 592 | $35,000 |
|  | 593 | $31,650 |
|  | 594 | $13,500 |
|  | 595 | $13,150 |
|  | 596 | $30,000 |
|  | 597 | $32,490 |
|  | 598 | $12,790 |
|  | 599 | $21,080 |
|  | 600 | $21,000 |
|  | 601 | $26,000 |
|  | 602 | $12,470 |
|  | 603 | $19,230 |
|  | 604 | $20,000 |
|  | 605 | $43,250 |
|  | 606 | $21,540 |
|  | 607 | $14,310 |
|  | 608 | $43,000 |
|  | 609 | $54,250 |
|  | 610 | $24,300 |
|  | 611 | $40,000 |
|  | 612 | $21,250 |
|  | 613 | $29,540 |
|  | 614 | $31,880 |
|  | 615 | $21,600 |
|  | 616 | $27,000 |
|  | 617 | $23,580 |
|  | 618 | $32,000 |
|  | 619 | $28,500 |
|  | 620 | $13,290 |
|  | 621 | $13,860 |
|  | 622 | $17,000 |
|  | 623 | $20,000 |
|  | 624 | $18,500 |
|  | 625 | $18,430 |
|  | 626 | $23,740 |
|  | 627 | $21,600 |
|  | 628 | $17,290 |
|  | 629 | $23,200 |
|  | 630 | $16,720 |
|  | 631 | $12,000 |
|  | 632 | $14,690 |
|  | 633 | $15,500 |
|  | 634 | $16,720 |
|  | 635 | $14,690 |
|  | 636 | $16,230 |
| SAI KUNG | 637 | $21,250 |
|  | 638 | $41,000 |
|  | 639 | $51,780 |
|  | 640 | $43,910 |
|  | 641 | $30,810 |
|  | 642 | $50,240 |
|  | 643 | $40,000 |
|  | 644 | $54,200 |
|  | 645 | $46,000 |
|  | 646 | $35,600 |
|  | 647 | $46,200 |
|  | 648 | $36,850 |
|  | 649 | $51,160 |
|  | 650 | $55,500 |
|  | 651 | $45,800 |
|  | 652 | $25,410 |
|  | 653 | $23,500 |
|  | 654 | $23,000 |
|  | 655 | $25,750 |
|  | 656 | $25,000 |
|  | 657 | $42,000 |
|  | 658 | $97,940 |
|  | 659 | $41,500 |
|  | 660 | $31,000 |
|  | 661 | $64,160 |
|  | 662 | $55,600 |
|  | 663 | $32,000 |
|  | 664 | $43,000 |
|  | 665 | $36,290 |
|  | 666 | $70,250 |
|  | 667 | $55,900 |
|  | 668 | $39,300 |
|  | 669 | $74,210 |
|  | 670 | $70,300 |
|  | 671 | $33,990 |
|  | 672 | $60,000 |
|  | 673 | $19,100 |
|  | 674 | $26,140 |
|  | 675 | $23,940 |
|  | 676 | $33,800 |
|  | 677 | $34,460 |
|  | 678 | $45,560 |
|  | 679 | $22,000 |
|  | 680 | $39,000 |
|  | 681 | $33,600 |
|  | 682 | $42,250 |
|  | 683 | $26,250 |
|  | 684 | $49,250 |
|  | 685 | $42,150 |
|  | 686 | $19,580 |
|  | 687 | $35,100 |
|  | 688 | $27,650 |
|  | 689 | $24,990 |
|  | 690 | $32,400 |
|  | 691 | $21,250 |
|  | 692 | $55,150 |
|  | 693 | $25,700 |
|  | 694 | $47,000 |
|  | 695 | $23,890 |
|  | 696 | $59,630 |
|  | 697 | $32,750 |
|  | 698 | $50,000 |
|  | 699 | $54,250 |
|  | 700 | $32,710 |
|  | 701 | $50,000 |
|  | 702 | $75,000 |
|  | 703 | $20,000 |
|  | 704 | $51,250 |
|  | 705 | $57,210 |
| SHA TIN | 706 | $92,750 |
|  | 707 | $48,400 |
|  | 708 | $35,060 |
|  | 709 | $80,500 |
|  | 710 | $44,350 |
|  | 711 | $89,210 |
|  | 712 | $48,000 |
|  | 713 | $90,000 |
|  | 714 | $61,290 |
|  | 715 | $63,940 |
|  | 716 | $31,590 |
|  | 717 | $27,000 |
|  | 718 | $15,890 |
|  | 719 | $23,000 |
|  | 720 | $35,000 |
|  | 721 | $34,500 |
|  | 722 | $30,000 |
|  | 723 | $17,000 |
|  | 724 | $44,160 |
|  | 725 | $17,000 |
|  | 726 | $38,250 |
|  | 727 | $40,000 |
|  | 728 | $13,930 |
|  | 729 | $84,800 |
|  | 730 | $65,300 |
|  | 731 | $17,500 |
|  | 732 | $17,490 |
|  | 733 | $13,090 |
|  | 734 | $30,100 |
|  | 735 | $25,000 |
|  | 736 | $27,000 |
|  | 737 | $30,200 |
|  | 738 | $32,000 |
|  | 739 | $53,000 |
|  | 740 | $13,940 |
|  | 741 | $50,000 |
|  | 742 | $60,000 |
|  | 743 | $13,620 |
|  | 744 | $74,160 |
|  | 745 | $43,200 |
|  | 746 | $21,250 |
|  | 747 | $27,740 |
|  | 748 | $37,500 |
|  | 749 | $36,000 |
|  | 750 | $30,000 |
|  | 751 | $25,000 |
|  | 752 | $28,810 |
|  | 753 | $56,160 |
|  | 754 | $35,400 |
|  | 755 | $23,000 |
|  | 756 | $30,740 |
|  | 757 | $15,000 |
|  | 758 | $44,600 |
|  | 759 | $67,200 |
|  | 760 | $26,290 |
|  | 761 | $23,750 |
|  | 762 | $27,460 |
|  | 763 | $27,000 |
|  | 764 | $25,000 |
|  | 765 | $41,000 |
|  | 766 | $30,850 |
|  | 767 | $49,000 |
|  | 768 | $54,160 |
|  | 769 | $56,510 |
|  | 770 | $36,200 |
|  | 771 | $62,240 |
|  | 772 | $49,470 |
|  | 773 | $47,510 |
|  | 774 | $47,000 |
|  | 775 | $72,900 |
|  | 776 | $29,000 |
|  | 777 | $27,900 |
|  | 778 | $23,000 |
|  | 779 | $58,000 |
|  | 780 | $58,750 |
|  | 781 | $46,500 |
|  | 782 | $44,000 |
|  | 783 | $26,250 |
|  | 784 | $30,340 |
|  | 785 | $15,200 |
|  | 786 | $16,670 |
|  | 787 | $22,870 |
|  | 788 | $28,750 |
|  | 789 | $36,000 |
|  | 790 | $100,210 |
|  | 791 | $31,000 |
|  | 792 | $95,900 |
|  | 793 | $55,490 |
|  | 794 | $27,000 |
|  | 795 | $30,000 |
|  | 796 | $28,250 |
|  | 797 | $65,000 |
|  | 798 | $15,490 |
|  | 799 | $30,170 |
|  | 800 | $32,900 |
|  | 801 | $15,900 |
|  | 802 | $40,000 |
|  | 803 | $53,160 |
|  | 804 | $23,600 |
|  | 805 | $14,530 |
|  | 806 | $31,250 |
|  | 807 | $18,000 |
|  | 808 | $13,000 |
|  | 809 | $22,580 |
|  | 810 | $48,750 |
| SHAM SHUI PO | 811 | $57,930 |
|  | 812 | $60,000 |
|  | 813 | $14,490 |
|  | 814 | $24,500 |
|  | 815 | $46,000 |
|  | 816 | $57,660 |
|  | 817 | $42,000 |
|  | 818 | $45,000 |
|  | 819 | $60,000 |
|  | 820 | $29,500 |
|  | 821 | $74,480 |
|  | 822 | $17,900 |
|  | 823 | $24,000 |
|  | 824 | $17,100 |
|  | 825 | $23,810 |
|  | 826 | $17,490 |
|  | 827 | $44,410 |
|  | 828 | $13,120 |
|  | 829 | $13,650 |
|  | 830 | $12,200 |
|  | 831 | $15,000 |
|  | 832 | $14,250 |
|  | 833 | $22,000 |
|  | 834 | $17,000 |
|  | 835 | $18,000 |
|  | 836 | $17,000 |
|  | 837 | $17,840 |
|  | 838 | $88,210 |
|  | 839 | $94,200 |
|  | 840 | $26,600 |
|  | 841 | $20,000 |
|  | 842 | $31,200 |
|  | 843 | $15,850 |
|  | 844 | $14,150 |
|  | 845 | $27,450 |
|  | 846 | $19,000 |
|  | 847 | $18,000 |
|  | 848 | $57,500 |
|  | 849 | $12,000 |
|  | 850 | $18,030 |
|  | 851 | $26,000 |
|  | 852 | $28,750 |
|  | 853 | $19,800 |
|  | 854 | $26,000 |
|  | 855 | $18,750 |
|  | 856 | $27,490 |
|  | 857 | $24,300 |
|  | 858 | $27,600 |
|  | 859 | $19,000 |
|  | 860 | $23,020 |
|  | 861 | $18,560 |
|  | 862 | $20,000 |
|  | 863 | $23,250 |
|  | 864 | $18,000 |
|  | 865 | $12,200 |
|  | 866 | $20,000 |
|  | 867 | $18,000 |
|  | 868 | $23,550 |
|  | 869 | $26,760 |
|  | 870 | $18,100 |
|  | 871 | $15,000 |
|  | 872 | $18,380 |
|  | 873 | $13,750 |
|  | 874 | $14,690 |
|  | 875 | $16,410 |
|  | 876 | $19,000 |
|  | 877 | $15,620 |
|  | 878 | $17,550 |
|  | 879 | $16,270 |
|  | 880 | $15,460 |
|  | 881 | $16,000 |
|  | 882 | $15,000 |
|  | 883 | $14,590 |
|  | 884 | $14,370 |
|  | 885 | $17,500 |
|  | 886 | $18,250 |
|  | 887 | $17,300 |
|  | 888 | $19,170 |
|  | 889 | $15,310 |
|  | 890 | $13,500 |
|  | 891 | $17,550 |
|  | 892 | $12,460 |
|  | 893 | $17,550 |
|  | 894 | $17,500 |
|  | 895 | $15,500 |
|  | 896 | $18,000 |
|  | 897 | $20,500 |
|  | 898 | $14,710 |
|  | 899 | $16,190 |
|  | 900 | $19,200 |
|  | 901 | $17,800 |
|  | 902 | $11,890 |
|  | 903 | $23,210 |
|  | 904 | $19,820 |
|  | 905 | $22,320 |
|  | 906 | $15,630 |
|  | 907 | $17,820 |
|  | 908 | $17,000 |
|  | 909 | $28,000 |
|  | 910 | $26,000 |
|  | 911 | $28,000 |
|  | 912 | $28,430 |
|  | 913 | $18,250 |
|  | 914 | $23,580 |
|  | 915 | $22,320 |
|  | 916 | $17,000 |
|  | 917 | $22,000 |
|  | 918 | $18,250 |
|  | 919 | $27,740 |
|  | 920 | $24,000 |
|  | 921 | $79,250 |
|  | 922 | $15,400 |
|  | 923 | $86,290 |
|  | 924 | $11,490 |
|  | 925 | $60,900 |
|  | 926 | $69,160 |
|  | 927 | $14,170 |
|  | 928 | $60,000 |
|  | 929 | $82,000 |
|  | 930 | $61,290 |
| SOUTHERN | 931 | $153,200 |
|  | 932 | $60,000 |
|  | 933 | $161,910 |
|  | 934 | $100,000 |
|  | 935 | $122,180 |
|  | 936 | $120,000 |
|  | 937 | $25,000 |
|  | 938 | $42,700 |
|  | 939 | $33,710 |
|  | 940 | $21,800 |
|  | 941 | $19,750 |
|  | 942 | $23,360 |
|  | 943 | $25,600 |
|  | 944 | $26,250 |
|  | 945 | $27,950 |
|  | 946 | $19,000 |
|  | 947 | $17,780 |
|  | 948 | $20,000 |
|  | 949 | $36,250 |
|  | 950 | $35,080 |
|  | 951 | $34,000 |
|  | 952 | $26,500 |
|  | 953 | $27,640 |
|  | 954 | $40,000 |
|  | 955 | $29,640 |
|  | 956 | $60,000 |
|  | 957 | $33,500 |
|  | 958 | $19,900 |
|  | 959 | $70,000 |
|  | 960 | $59,160 |
|  | 961 | $17,880 |
|  | 962 | $32,000 |
|  | 963 | $30,000 |
|  | 964 | $41,000 |
|  | 965 | $31,250 |
|  | 966 | $26,780 |
|  | 967 | $51,250 |
|  | 968 | $19,600 |
|  | 969 | $43,000 |
|  | 970 | $35,000 |
|  | 971 | $31,490 |
|  | 972 | $77,600 |
|  | 973 | $335,650 |
|  | 974 | $64,210 |
|  | 975 | $81,000 |
|  | 976 | $94,200 |
|  | 977 | $149,500 |
|  | 978 | $113,370 |
|  | 979 | $70,450 |
|  | 980 | $75,670 |
|  | 981 | $45,000 |
|  | 982 | $44,000 |
|  | 983 | $25,590 |
|  | 984 | $44,250 |
|  | 985 | $102,710 |
|  | 986 | $63,500 |
|  | 987 | $35,810 |
| TAI PO | 988 | $24,160 |
|  | 989 | $35,000 |
|  | 990 | $23,240 |
|  | 991 | $35,380 |
|  | 992 | $40,260 |
|  | 993 | $54,990 |
|  | 994 | $49,000 |
|  | 995 | $23,000 |
|  | 996 | $25,500 |
|  | 997 | $35,000 |
|  | 998 | $40,000 |
|  | 999 | $38,000 |
|  | 1000 | $115,000 |
|  | 1001 | $65,000 |
|  | 1002 | $29,650 |
|  | 1003 | $14,270 |
|  | 1004 | $14,590 |
|  | 1005 | $14,590 |
|  | 1006 | $14,270 |
|  | 1007 | $14,500 |
|  | 1008 | $14,270 |
|  | 1009 | $14,270 |
|  | 1010 | $26,080 |
|  | 1011 | $23,350 |
|  | 1012 | $30,000 |
|  | 1013 | $17,360 |
|  | 1014 | $30,000 |
|  | 1015 | $36,250 |
|  | 1016 | $39,630 |
|  | 1017 | $54,790 |
|  | 1018 | $56,000 |
|  | 1019 | $22,950 |
|  | 1020 | $30,750 |
|  | 1021 | $25,000 |
|  | 1022 | $27,700 |
|  | 1023 | $38,000 |
|  | 1024 | $35,000 |
|  | 1025 | $24,710 |
|  | 1026 | $44,990 |
|  | 1027 | $33,000 |
|  | 1028 | $22,650 |
|  | 1029 | $30,000 |
|  | 1030 | $28,000 |
|  | 1031 | $32,670 |
|  | 1032 | $28,210 |
|  | 1033 | $26,250 |
|  | 1034 | $29,600 |
|  | 1035 | $34,000 |
|  | 1036 | $30,000 |
|  | 1037 | $42,800 |
|  | 1038 | $44,000 |
|  | 1039 | $24,550 |
|  | 1040 | $16,650 |
|  | 1041 | $33,500 |
|  | 1042 | $26,910 |
|  | 1043 | $25,630 |
|  | 1044 | $18,490 |
|  | 1045 | $33,750 |
|  | 1046 | $29,550 |
|  | 1047 | $28,660 |
|  | 1048 | $39,720 |
|  | 1049 | $34,000 |
|  | 1050 | $29,990 |
|  | 1051 | $34,750 |
|  | 1052 | $33,840 |
|  | 1053 | $55,000 |
|  | 1054 | $64,750 |
|  | 1055 | $52,000 |
|  | 1056 | $55,370 |
|  | 1057 | $69,700 |
|  | 1058 | $35,000 |
|  | 1059 | $70,650 |
|  | 1060 | $61,320 |
|  | 1061 | $36,000 |
|  | 1062 | $36,450 |
|  | 1063 | $30,000 |
|  | 1064 | $43,500 |
|  | 1065 | $55,510 |
|  | 1066 | $55,000 |
| TSUEN WAN | 1067 | $18,500 |
|  | 1068 | $28,750 |
|  | 1069 | $18,800 |
|  | 1070 | $25,600 |
|  | 1071 | $32,250 |
|  | 1072 | $53,140 |
|  | 1073 | $34,300 |
|  | 1074 | $46,830 |
|  | 1075 | $28,300 |
|  | 1076 | $30,000 |
|  | 1077 | $35,000 |
|  | 1078 | $40,000 |
|  | 1079 | $43,000 |
|  | 1080 | $47,500 |
|  | 1081 | $58,490 |
|  | 1082 | $42,880 |
|  | 1083 | $49,200 |
|  | 1084 | $11,910 |
|  | 1085 | $19,900 |
|  | 1086 | $17,550 |
|  | 1087 | $13,500 |
|  | 1088 | $16,990 |
|  | 1089 | $31,250 |
|  | 1090 | $15,830 |
|  | 1091 | $26,500 |
|  | 1092 | $17,740 |
|  | 1093 | $19,580 |
|  | 1094 | $25,000 |
|  | 1095 | $21,340 |
|  | 1096 | $31,250 |
|  | 1097 | $35,000 |
|  | 1098 | $36,440 |
|  | 1099 | $15,000 |
|  | 1100 | $15,830 |
|  | 1101 | $19,580 |
|  | 1102 | $29,550 |
|  | 1103 | $37,660 |
|  | 1104 | $14,400 |
|  | 1105 | $55,000 |
|  | 1106 | $41,250 |
|  | 1107 | $18,000 |
|  | 1108 | $17,000 |
|  | 1109 | $25,000 |
|  | 1110 | $30,000 |
|  | 1111 | $45,000 |
|  | 1112 | $14,800 |
|  | 1113 | $19,750 |
|  | 1114 | $55,000 |
|  | 1115 | $44,800 |
|  | 1116 | $46,500 |
|  | 1117 | $47,700 |
|  | 1118 | $28,250 |
|  | 1119 | $40,750 |
|  | 1120 | $60,000 |
|  | 1121 | $49,000 |
|  | 1122 | $40,000 |
| TUEN MUN | 1123 | $19,660 |
|  | 1124 | $32,000 |
|  | 1125 | $14,330 |
|  | 1126 | $26,530 |
|  | 1127 | $31,650 |
|  | 1128 | $30,000 |
|  | 1129 | $30,000 |
|  | 1130 | $24,960 |
|  | 1131 | $36,000 |
|  | 1132 | $21,250 |
|  | 1133 | $23,410 |
|  | 1134 | $21,650 |
|  | 1135 | $45,450 |
|  | 1136 | $29,750 |
|  | 1137 | $30,000 |
|  | 1138 | $16,000 |
|  | 1139 | $11,750 |
|  | 1140 | $21,990 |
|  | 1141 | $29,150 |
|  | 1142 | $40,000 |
|  | 1143 | $45,660 |
|  | 1144 | $25,000 |
|  | 1145 | $20,000 |
|  | 1146 | $25,000 |
|  | 1147 | $9,000 |
|  | 1148 | $30,000 |
|  | 1149 | $40,000 |
|  | 1150 | $23,000 |
|  | 1151 | $29,000 |
|  | 1152 | $20,000 |
|  | 1153 | $18,080 |
|  | 1154 | $30,000 |
|  | 1155 | $22,330 |
|  | 1156 | $45,000 |
|  | 1157 | $27,720 |
|  | 1158 | $17,490 |
|  | 1159 | $30,000 |
|  | 1160 | $15,000 |
|  | 1161 | $17,000 |
|  | 1162 | $28,800 |
|  | 1163 | $45,660 |
|  | 1164 | $24,750 |
|  | 1165 | $31,500 |
|  | 1166 | $36,500 |
|  | 1167 | $18,500 |
|  | 1168 | $22,750 |
|  | 1169 | $13,650 |
|  | 1170 | $50,000 |
|  | 1171 | $32,210 |
|  | 1172 | $58,710 |
|  | 1173 | $17,250 |
|  | 1174 | $41,300 |
|  | 1175 | $73,490 |
|  | 1176 | $49,210 |
|  | 1177 | $30,000 |
|  | 1178 | $21,990 |
|  | 1179 | $40,000 |
|  | 1180 | $25,000 |
|  | 1181 | $25,500 |
|  | 1182 | $37,000 |
|  | 1183 | $41,000 |
|  | 1184 | $50,000 |
|  | 1185 | $20,000 |
|  | 1186 | $43,000 |
|  | 1187 | $48,000 |
|  | 1188 | $30,000 |
|  | 1189 | $55,000 |
|  | 1190 | $63,220 |
|  | 1191 | $48,350 |
|  | 1192 | $37,500 |
|  | 1193 | $40,250 |
|  | 1194 | $49,000 |
|  | 1195 | $24,050 |
|  | 1196 | $30,000 |
|  | 1197 | $28,000 |
|  | 1198 | $23,000 |
|  | 1199 | $28,000 |
|  | 1200 | $32,100 |
|  | 1201 | $26,000 |
|  | 1202 | $21,420 |
|  | 1203 | $35,000 |
| WAN CHAI | 1204 | $23,600 |
|  | 1205 | $23,250 |
|  | 1206 | $18,380 |
|  | 1207 | $20,000 |
|  | 1208 | $16,000 |
|  | 1209 | $20,800 |
|  | 1210 | $30,000 |
|  | 1211 | $18,000 |
|  | 1212 | $18,600 |
|  | 1213 | $32,000 |
|  | 1214 | $30,800 |
|  | 1215 | $50,000 |
|  | 1216 | $29,650 |
|  | 1217 | $23,250 |
|  | 1218 | $38,000 |
|  | 1219 | $34,000 |
|  | 1220 | $63,710 |
|  | 1221 | $74,250 |
|  | 1222 | $20,800 |
|  | 1223 | $19,500 |
|  | 1224 | $35,000 |
|  | 1225 | $30,800 |
|  | 1226 | $14,500 |
|  | 1227 | $21,250 |
|  | 1228 | $19,500 |
|  | 1229 | $42,650 |
|  | 1230 | $42,000 |
|  | 1231 | $20,000 |
|  | 1232 | $30,000 |
|  | 1233 | $29,000 |
|  | 1234 | $18,600 |
|  | 1235 | $27,950 |
|  | 1236 | $21,250 |
|  | 1237 | $14,200 |
|  | 1238 | $42,210 |
|  | 1239 | $27,000 |
|  | 1240 | $70,800 |
|  | 1241 | $76,210 |
|  | 1242 | $50,000 |
|  | 1243 | $30,000 |
|  | 1244 | $100,000 |
|  | 1245 | $93,750 |
|  | 1246 | $101,200 |
|  | 1247 | $69,000 |
|  | 1248 | $73,000 |
|  | 1249 | $34,160 |
|  | 1250 | $41,750 |
|  | 1251 | $56,640 |
|  | 1252 | $44,500 |
|  | 1253 | $35,200 |
|  | 1254 | $51,000 |
|  | 1255 | $81,800 |
|  | 1256 | $69,320 |
|  | 1257 | $60,000 |
|  | 1258 | $96,000 |
|  | 1259 | $40,000 |
|  | 1260 | $43,490 |
|  | 1261 | $77,000 |
|  | 1262 | $90,450 |
|  | 1263 | $54,830 |
|  | 1264 | $99,000 |
|  | 1265 | $20,270 |
|  | 1266 | $18,000 |
|  | 1267 | $50,230 |
|  | 1268 | $18,800 |
|  | 1269 | $42,250 |
|  | 1270 | $34,500 |
|  | 1271 | $22,000 |
|  | 1272 | $19,000 |
|  | 1273 | $34,600 |
|  | 1274 | $45,000 |
|  | 1275 | $50,210 |
|  | 1276 | $97,700 |
|  | 1277 | $115,550 |
|  | 1278 | $114,160 |
|  | 1279 | $86,000 |
|  | 1280 | $148,200 |
|  | 1281 | $83,470 |
| WONG TAI SIN | 1282 | $16,000 |
|  | 1283 | $23,090 |
|  | 1284 | $27,000 |
|  | 1285 | $22,300 |
|  | 1286 | $24,750 |
|  | 1287 | $26,000 |
|  | 1288 | $22,500 |
|  | 1289 | $22,330 |
|  | 1290 | $15,000 |
|  | 1291 | $31,850 |
|  | 1292 | $25,000 |
|  | 1293 | $34,000 |
|  | 1294 | $70,000 |
|  | 1295 | $35,000 |
|  | 1296 | $33,500 |
|  | 1297 | $20,750 |
|  | 1298 | $30,000 |
|  | 1299 | $55,200 |
|  | 1300 | $15,000 |
|  | 1301 | $28,000 |
|  | 1302 | $21,590 |
|  | 1303 | $20,600 |
|  | 1304 | $19,140 |
|  | 1305 | $29,920 |
|  | 1306 | $22,510 |
|  | 1307 | $20,000 |
|  | 1308 | $16,000 |
|  | 1309 | $28,830 |
|  | 1310 | $28,200 |
|  | 1311 | $19,270 |
|  | 1312 | $31,000 |
|  | 1313 | $32,310 |
|  | 1314 | $23,780 |
|  | 1315 | $20,490 |
|  | 1316 | $21,170 |
|  | 1317 | $17,500 |
|  | 1318 | $16,990 |
|  | 1319 | $16,150 |
|  | 1320 | $21,000 |
|  | 1321 | $21,310 |
|  | 1322 | $15,960 |
|  | 1323 | $38,620 |
|  | 1324 | $15,750 |
|  | 1325 | $15,420 |
|  | 1326 | $13,800 |
|  | 1327 | $5,900 |
|  | 1328 | $14,540 |
|  | 1329 | $15,000 |
|  | 1330 | $16,500 |
|  | 1331 | $20,400 |
|  | 1332 | $18,800 |
|  | 1333 | $30,000 |
|  | 1334 | $19,880 |
|  | 1335 | $30,000 |
|  | 1336 | $44,210 |
|  | 1337 | $15,290 |
|  | 1338 | $56,410 |
|  | 1339 | $21,450 |
|  | 1340 | $19,500 |
|  | 1341 | $19,450 |
|  | 1342 | $22,250 |
|  | 1343 | $41,000 |
|  | 1344 | $30,000 |
|  | 1345 | $37,200 |
|  | 1346 | $30,360 |
|  | 1347 | $31,050 |
|  | 1348 | $13,000 |
|  | 1349 | $45,460 |
| YAU TSIM MONG | 1350 | $20,000 |
|  | 1351 | $16,780 |
|  | 1352 | $17,210 |
|  | 1353 | $21,430 |
|  | 1354 | $23,000 |
|  | 1355 | $69,160 |
|  | 1356 | $29,820 |
|  | 1357 | $22,000 |
|  | 1358 | $14,150 |
|  | 1359 | $38,750 |
|  | 1360 | $23,660 |
|  | 1361 | $23,130 |
|  | 1362 | $15,630 |
|  | 1363 | $32,540 |
|  | 1364 | $16,650 |
|  | 1365 | $19,210 |
|  | 1366 | $22,480 |
|  | 1367 | $21,000 |
|  | 1368 | $19,500 |
|  | 1369 | $79,750 |
|  | 1370 | $30,000 |
|  | 1371 | $12,490 |
|  | 1372 | $18,920 |
|  | 1373 | $20,000 |
|  | 1374 | $19,730 |
|  | 1375 | $14,290 |
|  | 1376 | $20,000 |
|  | 1377 | $41,000 |
|  | 1378 | $39,000 |
|  | 1379 | $12,490 |
|  | 1380 | $12,790 |
|  | 1381 | $14,700 |
|  | 1382 | $25,000 |
|  | 1383 | $15,500 |
|  | 1384 | $15,500 |
|  | 1385 | $16,130 |
|  | 1386 | $19,660 |
|  | 1387 | $16,500 |
|  | 1388 | $17,630 |
|  | 1389 | $16,750 |
|  | 1390 | $16,200 |
|  | 1391 | $16,200 |
|  | 1392 | $17,490 |
|  | 1393 | $19,000 |
|  | 1394 | $17,630 |
|  | 1395 | $20,000 |
|  | 1396 | $20,000 |
|  | 1397 | $16,300 |
|  | 1398 | $34,000 |
|  | 1399 | $15,900 |
|  | 1400 | $18,630 |
|  | 1401 | $20,000 |
|  | 1402 | $12,920 |
|  | 1403 | $16,490 |
|  | 1404 | $19,830 |
|  | 1405 | $30,600 |
|  | 1406 | $21,250 |
|  | 1407 | $24,000 |
|  | 1408 | $17,850 |
|  | 1409 | $19,500 |
|  | 1410 | $16,490 |
|  | 1411 | $20,000 |
|  | 1412 | $31,270 |
|  | 1413 | $38,750 |
|  | 1414 | $26,000 |
|  | 1415 | $20,160 |
|  | 1416 | $13,500 |
|  | 1417 | $16,100 |
|  | 1418 | $14,850 |
|  | 1419 | $17,380 |
|  | 1420 | $15,000 |
|  | 1421 | $20,500 |
|  | 1422 | $20,000 |
|  | 1423 | $15,000 |
|  | 1424 | $21,840 |
|  | 1425 | $15,500 |
|  | 1426 | $19,800 |
|  | 1427 | $16,400 |
|  | 1428 | $16,200 |
|  | 1429 | $18,740 |
|  | 1430 | $16,900 |
|  | 1431 | $21,290 |
|  | 1432 | $21,590 |
|  | 1433 | $16,480 |
|  | 1434 | $17,000 |
|  | 1435 | $20,000 |
|  | 1436 | $19,900 |
|  | 1437 | $19,900 |
|  | 1438 | $24,300 |
|  | 1439 | $18,800 |
|  | 1440 | $17,370 |
|  | 1441 | $18,250 |
|  | 1442 | $18,740 |
|  | 1443 | $16,640 |
|  | 1444 | $34,000 |
|  | 1445 | $40,000 |
|  | 1446 | $18,490 |
|  | 1447 | $100,000 |
|  | 1448 | $87,000 |
|  | 1449 | $17,800 |
|  | 1450 | $20,250 |
|  | 1451 | $16,500 |
|  | 1452 | $18,170 |
|  | 1453 | $19,640 |
|  | 1454 | $26,000 |
|  | 1455 | $19,640 |
|  | 1456 | $17,500 |
|  | 1457 | $18,050 |
|  | 1458 | $34,250 |
|  | 1459 | $17,500 |
|  | 1460 | $23,200 |
|  | 1461 | $17,800 |
|  | 1462 | $18,500 |
|  | 1463 | $19,420 |
|  | 1464 | $21,840 |
|  | 1465 | $14,000 |
|  | 1466 | $13,000 |
|  | 1467 | $13,740 |
|  | 1468 | $14,850 |
|  | 1469 | $15,880 |
|  | 1470 | $13,050 |
|  | 1471 | $16,990 |
|  | 1472 | $19,000 |
|  | 1473 | $89,160 |
|  | 1474 | $86,950 |
|  | 1475 | $46,750 |
|  | 1476 | $67,000 |
|  | 1477 | $21,000 |
|  | 1478 | $35,000 |
|  | 1479 | $35,000 |
|  | 1480 | $55,000 |
|  | 1481 | $84,000 |
|  | 1482 | $83,000 |
|  | 1483 | $46,750 |
|  | 1484 | $65,000 |
| YUEN LONG | 1485 | $28,500 |
|  | 1486 | $21,250 |
|  | 1487 | $22,490 |
|  | 1488 | $28,940 |
|  | 1489 | $20,180 |
|  | 1490 | $30,150 |
|  | 1491 | $28,160 |
|  | 1492 | $27,000 |
|  | 1493 | $26,000 |
|  | 1494 | $17,670 |
|  | 1495 | $30,490 |
|  | 1496 | $24,850 |
|  | 1497 | $27,250 |
|  | 1498 | $10,250 |
|  | 1499 | $22,000 |
|  | 1500 | $12,750 |
|  | 1501 | $14,000 |
|  | 1502 | $17,200 |
|  | 1503 | $28,780 |
|  | 1504 | $31,800 |
|  | 1505 | $19,250 |
|  | 1506 | $21,490 |
|  | 1507 | $25,000 |
|  | 1508 | $18,500 |
|  | 1509 | $20,670 |
|  | 1510 | $30,000 |
|  | 1511 | $20,000 |
|  | 1512 | $23,500 |
|  | 1513 | $13,000 |
|  | 1514 | $31,830 |
|  | 1515 | $14,500 |
|  | 1516 | $12,000 |
|  | 1517 | $12,250 |
|  | 1518 | $25,000 |
|  | 1519 | $20,000 |
|  | 1520 | $23,000 |
|  | 1521 | $21,000 |
|  | 1522 | $21,250 |
|  | 1523 | $31,000 |
|  | 1524 | $26,740 |
|  | 1525 | $23,740 |
|  | 1526 | $13,950 |
|  | 1527 | $25,000 |
|  | 1528 | $28,750 |
|  | 1529 | $31,250 |
|  | 1530 | $38,000 |
|  | 1531 | $40,000 |
|  | 1532 | $26,000 |
|  | 1533 | $23,000 |
|  | 1534 | $26,000 |
|  | 1535 | $20,000 |
|  | 1536 | $25,460 |
|  | 1537 | $15,990 |
|  | 1538 | $53,000 |
|  | 1539 | $21,250 |
|  | 1540 | $18,750 |
|  | 1541 | $23,750 |
|  | 1542 | $24,000 |
|  | 1543 | $27,750 |
|  | 1544 | $25,000 |
|  | 1545 | $24,490 |
|  | 1546 | $19,200 |
|  | 1547 | $25,000 |
|  | 1548 | $18,720 |
|  | 1549 | $15,500 |
|  | 1550 | $20,000 |
|  | 1551 | $21,160 |
|  | 1552 | $14,380 |
|  | 1553 | $21,250 |
|  | 1554 | $11,480 |
|  | 1555 | $14,750 |
|  | 1556 | $13,050 |
|  | 1557 | $15,000 |
|  | 1558 | $24,200 |
|  | 1559 | $18,500 |
|  | 1560 | $19,700 |
|  | 1561 | $25,710 |
|  | 1562 | $21,250 |
|  | 1563 | $28,160 |
|  | 1564 | $20,300 |
|  | 1565 | $21,650 |
|  | 1566 | $19,200 |
|  | 1567 | $39,250 |
|  | 1568 | $20,450 |
|  | 1569 | $34,000 |
|  | 1570 | $23,500 |
|  | 1571 | $39,900 |
|  | 1572 | $24,490 |
|  | 1573 | $24,160 |
|  | 1574 | $26,250 |
|  | 1575 | $15,000 |
|  | 1576 | $34,500 |
|  | 1577 | $13,240 |
|  | 1578 | $20,960 |
|  | 1579 | $16,500 |
|  | 1580 | $28,500 |
|  | 1581 | $47,000 |
|  | 1582 | $21,750 |
|  | 1583 | $19,000 |
|  | 1584 | $18,000 |
|  | 1585 | $27,380 |
|  | 1586 | $18,000 |
|  | 1587 | $43,000 |
|  | 1588 | $31,250 |
|  | 1589 | $38,460 |
|  | 1590 | $25,350 |
|  | 1591 | $35,500 |
|  | 1592 | $23,000 |
|  | 1593 | $46,210 |
|  | 1594 | $39,000 |
|  | 1595 | $30,500 |
|  | 1596 | $19,060 |
|  | 1597 | $35,000 |
|  | 1598 | $25,450 |
|  | 1599 | $42,710 |
|  | 1600 | $20,000 |
|  | 1601 | $21,650 |
|  | 1602 | $31,150 |
|  | 1603 | $17,410 |
|  | 1604 | $25,500 |
|  | 1605 | $21,750 |
|  | 1606 | $19,750 |
|  | 1607 | $23,500 |
|  | 1608 | $50,000 |
|  | 1609 | $31,290 |
|  | 1610 | $20,000 |
|  | 1611 | $18,250 |
|  | 1612 | $23,200 |
|  | 1613 | $25,780 |
|  | 1614 | $53,040 |
|  | 1615 | $19,750 |
|  | 1616 | $18,000 |
|  | 1617 | $29,000 |
|  | 1618 | $76,000 |
|  | 1619 | $27,000 |
|  | 1620 | $27,350 |
|  | 1621 | $47,000 |
|  | 1622 | $14,500 |

S2 Table. Normalized green and blue spaces accessibility score and COVID-19 infection risk score by LSBG

| **District** | **LSBG  Objective ID** | **Normalizaed Green  and Blue Spaces  Accessibility Score** | **Normalized  COVID-19 Infection Risk Score** |
| --- | --- | --- | --- |
| CENTRAL  & WESTERN | 1 | 0.00221748 | 0.023443397 |
|  | 2 | 0.000104534 | 0 |
|  | 3 | 5.2113E-05 | 0.091417737 |
|  | 4 | 5.1952E-05 | 0 |
|  | 5 | 6.38323E-05 | 0.051154089 |
|  | 6 | 3.65357E-05 | 0.055952628 |
|  | 7 | 6.68405E-05 | 0.11999908 |
|  | 8 | 4.34951E-05 | 0.089368551 |
|  | 9 | 2.88275E-05 | 0.116774521 |
|  | 10 | 1.29232E-05 | 0.15247614 |
|  | 11 | 0.006057909 | 0.03464338 |
|  | 12 | 1.13212E-05 | 0.09080685 |
|  | 13 | 1.08211E-05 | 0.102217188 |
|  | 14 | 9.61126E-05 | 0.113567002 |
|  | 15 | 5.20574E-05 | 0.127086569 |
|  | 16 | 6.49608E-05 | 0.081619444 |
|  | 17 | 0.000154718 | 0.113029028 |
|  | 18 | 5.45759E-05 | 0.033565199 |
|  | 19 | 0.000226743 | 0.075631792 |
|  | 20 | 5.49881E-05 | 0.125461507 |
|  | 21 | 0.000154902 | 0.008302732 |
|  | 22 | 4.56426E-06 | 0.075981183 |
|  | 23 | 4.55047E-05 | 0.175533196 |
|  | 24 | 3.92814E-05 | 0.259531812 |
|  | 25 | 2.25812E-05 | 0.189357253 |
|  | 26 | 2.86416E-05 | 0.292374223 |
|  | 27 | 1.84356E-05 | 0.307891266 |
|  | 28 | 8.39761E-06 | 0.237599532 |
|  | 29 | 2.75475E-05 | 0.273997429 |
|  | 30 | 1.19504E-05 | 0.169060853 |
|  | 31 | 5.18339E-06 | 0.11738423 |
|  | 32 | 2.469E-06 | 0.185699708 |
|  | 33 | 1.01395E-05 | 0.235542776 |
|  | 34 | 1.96538E-05 | 0 |
|  | 35 | 1.444E-05 | 0.289035181 |
|  | 36 | 5.55989E-06 | 0.133721454 |
|  | 37 | 1.46874E-05 | 0.110617091 |
|  | 38 | 4.26235E-06 | 0.118255315 |
|  | 39 | 6.43798E-06 | 0.322392557 |
|  | 40 | 3.45233E-06 | 0.359274873 |
|  | 41 | 5.86883E-06 | 0 |
|  | 42 | 2.98515E-06 | 0.167698816 |
|  | 43 | 5.98914E-06 | 0 |
|  | 44 | 8.11906E-06 | 0.108135917 |
|  | 45 | 1.02226E-06 | 0.303303156 |
|  | 46 | 1.02394E-05 | 0.35565113 |
|  | 47 | 7.11991E-06 | 0.231838162 |
|  | 48 | 1.74387E-06 | 0.323090385 |
|  | 49 | 1.62934E-06 | 0.383981479 |
|  | 50 | 2.56268E-06 | 0.193605888 |
|  | 51 | 9.73785E-07 | 0.375253098 |
|  | 52 | 1.94506E-06 | 0.413777269 |
|  | 53 | 6.28176E-07 | 0.433334906 |
|  | 54 | 2.26982E-06 | 0.386272816 |
|  | 55 | 9.98651E-07 | 0.424012159 |
|  | 56 | 1.88578E-06 | 0.449485235 |
|  | 57 | 2.45443E-06 | 0.412766784 |
|  | 58 | 5.5408E-07 | 0.493255423 |
|  | 59 | 7.64963E-06 | 0.201019605 |
|  | 60 | 8.31372E-06 | 0.287289147 |
|  | 61 | 6.90466E-07 | 0.338501511 |
|  | 62 | 1.46181E-06 | 0.473717724 |
|  | 63 | 1.60874E-06 | 0.472094026 |
|  | 64 | 1.20388E-05 | 0.081644605 |
|  | 65 | 1.08955E-05 | 0 |
|  | 66 | 1.23324E-06 | 0.154433826 |
|  | 67 | 8.25972E-06 | 0 |
|  | 68 | 1.39603E-05 | 0.184876518 |
|  | 69 | 1.27142E-05 | 0.17999189 |
|  | 70 | 4.73E-05 | 0.275233434 |
|  | 71 | 9.86846E-07 | 0.254085457 |
|  | 72 | 3.38049E-06 | 0.170635763 |
|  | 73 | 0.000273782 | 0.270514149 |
|  | 74 | 0.006163522 | 0.052712786 |
|  | 75 | 2.54661E-06 | 0.153490183 |
|  | 76 | 3.89539E-06 | 0.293733278 |
|  | 77 | 2.80544E-05 | 0.32719308 |
|  | 78 | 6.53702E-05 | 0.242303524 |
|  | 79 | 7.09537E-05 | 0.248398583 |
|  | 80 | 0.000509072 | 0.1196742 |
|  | 81 | 8.87918E-05 | 0.29436974 |
|  | 82 | 1.77906E-05 | 0.361076843 |
|  | 83 | 2.97109E-06 | 0.430569082 |
|  | 84 | 0.000152808 | 0.228834804 |
|  | 85 | 1.00435E-05 | 0.315027019 |
|  | 86 | 8.75703E-06 | 0.368792157 |
|  | 87 | 1.99981E-06 | 0.417329542 |
|  | 88 | 1.20009E-06 | 0.184312442 |
|  | 89 | 3.50078E-05 | 0.437314075 |
|  | 90 | 7.32584E-05 | 0.339727381 |
|  | 91 | 9.89945E-05 | 0.283568433 |
|  | 92 | 0.000113784 | 0.176563792 |
|  | 93 | 1.82801E-06 | 0.235628273 |
|  | 94 | 0.000114236 | 0.103316291 |
|  | 95 | 6.66376E-05 | 0.222860364 |
|  | 96 | 0.00054247 | 0.144612923 |
|  | 97 | 0.00013238 | 0.174981115 |
|  | 98 | 0.000328771 | 0.11442389 |
|  | 99 | 0.000879856 | 0.052457366 |
|  | 100 | 0.092641756 | 0.02523086 |
|  | 101 | 0.00310077 | 0.008752511 |
|  | 102 | 0.00144218 | 0.008086137 |
| EASTERN | 103 | 1.43167E-05 | 0.32409178 |
|  | 104 | 0.000465629 | 0.036739692 |
|  | 105 | 2.07818E-06 | 0.071043188 |
|  | 106 | 1.17598E-06 | 0.177411193 |
|  | 107 | 1.26087E-07 | 0.11452517 |
|  | 108 | 2.53054E-06 | 0.163863155 |
|  | 109 | 3.05473E-06 | 0.113943423 |
|  | 110 | 5.61892E-06 | 0.215450706 |
|  | 111 | 5.5388E-06 | 0.124099863 |
|  | 112 | 2.2015E-06 | 0 |
|  | 113 | 0 | 0.265238357 |
|  | 114 | 4.82211E-05 | 0.193903906 |
|  | 115 | 3.38622E-05 | 0.248405352 |
|  | 116 | 4.75273E-05 | 0.179231601 |
|  | 117 | 4.65199E-05 | 0 |
|  | 118 | 3.7039E-05 | 0.084011926 |
|  | 119 | 2.80828E-05 | 0 |
|  | 120 | 1.62128E-05 | 0.081908547 |
|  | 121 | 6.89225E-05 | 0 |
|  | 122 | 4.10715E-05 | 0.090123339 |
|  | 123 | 0 | 0.235229283 |
|  | 124 | 6.16993E-05 | 0.165692706 |
|  | 125 | 0.000291551 | 0.23100497 |
|  | 126 | 0.000276234 | 0.1849467 |
|  | 127 | 7.15316E-05 | 0.155962449 |
|  | 128 | 0.000674403 | 0.099715019 |
|  | 129 | 0.000288124 | 0.089242023 |
|  | 130 | 0 | 0.18485154 |
|  | 131 | 0 | 0.268086062 |
|  | 132 | 0 | 0.264039503 |
|  | 133 | 0 | 0.283825938 |
|  | 134 | 0 | 0.248889264 |
|  | 135 | 0 | 0.289465402 |
|  | 136 | 0 | 0.285076775 |
|  | 137 | 0 | 0.277449672 |
|  | 138 | 0 | 0.264799199 |
|  | 139 | 8.65104E-06 | 0 |
|  | 140 | 0 | 0.296989774 |
|  | 141 | 0 | 0.247724577 |
|  | 142 | 0 | 0.269883968 |
|  | 143 | 4.64172E-05 | 0.226776769 |
|  | 144 | 0 | 0.203477059 |
|  | 145 | 1.51347E-05 | 0.179480704 |
|  | 146 | 1.06634E-05 | 0.298048638 |
|  | 147 | 4.08686E-05 | 0.299645633 |
|  | 148 | 0.000108859 | 0 |
|  | 149 | 0.000144279 | 0 |
|  | 150 | 0.000196698 | 0.263604895 |
|  | 151 | 0.000145269 | 0.155739913 |
|  | 152 | 6.71477E-05 | 0.191452498 |
|  | 153 | 2.63163E-05 | 0.097119258 |
|  | 154 | 0.000242397 | 0.280638483 |
|  | 155 | 5.2815E-05 | 0.138539797 |
|  | 156 | 8.15882E-05 | 0 |
|  | 157 | 0.000118512 | 0.284793284 |
|  | 158 | 0 | 0.249877604 |
|  | 159 | 0.000262779 | 0.070362302 |
|  | 160 | 0.000197309 | 0 |
|  | 161 | 0.000494969 | 0.109907282 |
|  | 162 | 3.10521E-06 | 0.14563574 |
|  | 163 | 0.000800969 | 0.097396773 |
|  | 164 | 4.9383E-05 | 0.107444253 |
|  | 165 | 0.000305001 | 0.135742518 |
|  | 166 | 0.000215822 | 0.072827566 |
|  | 167 | 0.000121183 | 0.15842275 |
|  | 168 | 1.18429E-05 | 0.179404114 |
|  | 169 | 0.000104928 | 0.162647328 |
|  | 170 | 0.000175648 | 0 |
|  | 171 | 0.000723164 | 0.117627512 |
|  | 172 | 4.36832E-05 | 0.1522809 |
|  | 173 | 7.02723E-06 | 0.167213051 |
|  | 174 | 6.45933E-06 | 0.179326706 |
|  | 175 | 2.14524E-06 | 0.191719382 |
|  | 176 | 7.96057E-06 | 0.167556372 |
|  | 177 | 7.88397E-06 | 0.147195398 |
|  | 178 | 4.51226E-06 | 0 |
|  | 179 | 0.050946329 | 0.073505466 |
|  | 180 | 3.99506E-05 | 0.122229516 |
|  | 181 | 2.09026E-05 | 0.168948544 |
|  | 182 | 1.10346E-05 | 0.189827847 |
|  | 183 | 7.10534E-06 | 0.161449573 |
|  | 184 | 0 | 0 |
|  | 185 | 4.35754E-06 | 0 |
|  | 186 | 5.22772E-05 | 0.132274718 |
|  | 187 | 1.27946E-06 | 0.097318096 |
|  | 188 | 9.85088E-07 | 0.102657343 |
|  | 189 | 1.56429E-06 | 0.089660328 |
|  | 190 | 2.83822E-07 | 0.126329832 |
|  | 191 | 1.6321E-06 | 0.156338195 |
|  | 192 | 8.15448E-06 | 0.167623343 |
|  | 193 | 1.63763E-06 | 0.182909968 |
|  | 194 | 2.69447E-05 | 0.178169125 |
|  | 195 | 7.70765E-06 | 0.168147739 |
|  | 196 | 4.82924E-06 | 0.144781855 |
|  | 197 | 4.69838E-06 | 0 |
|  | 198 | 4.01118E-07 | 0.13140942 |
|  | 199 | 2.83822E-08 | 0.16816616 |
|  | 200 | 1.72137E-05 | 0.17367228 |
|  | 201 | 4.34609E-05 | 0.089267444 |
|  | 202 | 2.41123E-08 | 0.103044259 |
|  | 203 | 1.21169E-05 | 0 |
|  | 204 | 3.91825E-08 | 0.207318301 |
|  | 205 | 7.15984E-06 | 0 |
|  | 206 | 7.52756E-06 | 0.191234814 |
|  | 207 | 9.20914E-06 | 0.163335111 |
|  | 208 | 5.49057E-06 | 0.046107389 |
|  | 209 | 3.60353E-06 | 0.172249124 |
|  | 210 | 2.5451E-05 | 0.064596112 |
|  | 211 | 0.000114448 | 0.179991087 |
|  | 212 | 0.000101717 | 0.067310179 |
|  | 213 | 0 | 0.105265173 |
|  | 214 | 1.50001E-05 | 0.102160194 |
|  | 215 | 1.41941E-05 | 0.09637637 |
|  | 216 | 5.92133E-06 | 0.172752367 |
|  | 217 | 8.60618E-05 | 0 |
|  | 218 | 8.60618E-05 | 0.124096841 |
|  | 219 | 0.00051958 | 0.064657312 |
|  | 220 | 4.96311E-07 | 0.165993514 |
|  | 221 | 2.74177E-06 | 0.194897256 |
|  | 222 | 0 | 0.185710573 |
|  | 223 | 5.695E-05 | 0.097845185 |
|  | 224 | 9.29328E-09 | 0.193376473 |
|  | 225 | 0.000418572 | 0.028628847 |
|  | 226 | 7.34872E-06 | 0.094837339 |
|  | 227 | 3.42847E-06 | 0.058811732 |
|  | 228 | 0.000105661 | 0.062959036 |
|  | 229 | 4.12423E-05 | 0.086554261 |
|  | 230 | 7.01392E-06 | 0.124644972 |
|  | 231 | 4.17469E-06 | 0.104553115 |
|  | 232 | 1.2021E-06 | 0.096280093 |
|  | 233 | 5.72308E-05 | 0.122131246 |
|  | 234 | 4.20333E-06 | 0.11738077 |
|  | 235 | 0.002224353 | 0.045408556 |
|  | 236 | 7.89251E-06 | 0.124852935 |
|  | 237 | 0.00183301 | 0.025100757 |
|  | 238 | 1.27418E-05 | 0.114099537 |
|  | 239 | 0.000275775 | 0.096727037 |
|  | 240 | 0.000623521 | 0.103164384 |
|  | 241 | 2.15993E-05 | 0.101146938 |
|  | 242 | 0.000572514 | 0.028871769 |
|  | 243 | 0.000247084 | 0.101999567 |
|  | 244 | 0.001502648 | 0.074991046 |
|  | 245 | 0.000144929 | 0.112031583 |
|  | 246 | 0.000120045 | 0.16451313 |
|  | 247 | 0.000383674 | 0.127987416 |
|  | 248 | 0.000345428 | 0.066116734 |
|  | 249 | 1.66234E-05 | 0.074922353 |
|  | 250 | 1.47098E-05 | 0.099381988 |
|  | 251 | 1.40052E-06 | 0.096303512 |
|  | 252 | 7.81969E-05 | 0.085277643 |
|  | 253 | 0.00028659 | 0.097854328 |
|  | 254 | 6.48118E-06 | 0.108438749 |
|  | 255 | 0.004183685 | 0.041204934 |
| ISLANDS | 256 | 0.227570128 | 0.000819145 |
|  | 257 | 0.007933574 | 0.002291947 |
|  | 258 | 0.007996004 | 0.001103117 |
|  | 259 | 0.007933574 | 0.003230254 |
|  | 260 | 0 | 0.003385907 |
|  | 261 | 0 | 0 |
|  | 262 | 0 | 0.008696291 |
|  | 263 | 0 | 0.003125467 |
|  | 264 | 0 | 0.009142633 |
|  | 265 | 0 | 0.005640886 |
|  | 266 | 0 | 0.009134594 |
|  | 267 | 0.234288764 | 6.23135E-05 |
|  | 268 | 0.114562082 | 0.000642601 |
|  | 269 | 0.012647246 | 0.001012359 |
|  | 270 | 0.816310496 | 3.14337E-05 |
|  | 271 | 0.013705927 | 0.001173366 |
|  | 272 | 0.004304741 | 0.00187774 |
|  | 273 | 0.144676677 | 0.016223946 |
|  | 274 | 1.41115E-05 | 0.050760585 |
|  | 275 | 0.045437703 | 0.002504504 |
|  | 276 | 1.70512E-05 | 0.063472152 |
|  | 277 | 0.272518258 | 0.000174496 |
|  | 278 | 0.001029148 | 0.004944024 |
|  | 279 | 0.030866128 | 0.001453454 |
|  | 280 | 0.023132189 | 0.005212022 |
|  | 281 | 0.001108096 | 0 |
|  | 282 | 0.001108096 | 0.005705582 |
|  | 283 | 0.001108096 | 0.004686715 |
|  | 284 | 0.001108096 | 0.004300364 |
| KOWLOON CITY | 285 | 0 | 0.317180772 |
|  | 286 | 0 | 0.263417373 |
|  | 287 | 0 | 0.13585886 |
|  | 288 | 1.47612E-06 | 0.146855822 |
|  | 289 | 3.18508E-06 | 0.098462348 |
|  | 290 | 6.4744E-06 | 0.069217752 |
|  | 291 | 5.44755E-05 | 0.291866505 |
|  | 292 | 4.54027E-05 | 0.338039963 |
|  | 293 | 9.55877E-06 | 0.14230509 |
|  | 294 | 2.86128E-05 | 0.141524711 |
|  | 295 | 1.29164E-05 | 0.172897228 |
|  | 296 | 1.20303E-05 | 0.199727147 |
|  | 297 | 6.34605E-06 | 0.251189169 |
|  | 298 | 1.19426E-05 | 0 |
|  | 299 | 1.84946E-05 | 0.330533359 |
|  | 300 | 1.15541E-05 | 0.337900687 |
|  | 301 | 6.41186E-06 | 0.216907127 |
|  | 302 | 6.64721E-06 | 0.68946448 |
|  | 303 | 4.80965E-06 | 0.581359373 |
|  | 304 | 0 | 0.488834138 |
|  | 305 | 2.67747E-06 | 0.706025714 |
|  | 306 | 2.16719E-05 | 0.539851879 |
|  | 307 | 2.76892E-05 | 0.41948284 |
|  | 308 | 2.73881E-05 | 0.359264273 |
|  | 309 | 8.43227E-06 | 0.219107585 |
|  | 310 | 0.000164004 | 0.224245197 |
|  | 311 | 1.8044E-05 | 0.20711795 |
|  | 312 | 3.21962E-05 | 0.234110327 |
|  | 313 | 2.13753E-05 | 0.229434094 |
|  | 314 | 2.75257E-06 | 0.221943804 |
|  | 315 | 0.000106991 | 0.251938106 |
|  | 316 | 0 | 0.178641726 |
|  | 317 | 1.45678E-08 | 0 |
|  | 318 | 7.28392E-09 | 0.272729644 |
|  | 319 | 1.95912E-08 | 0.282084616 |
|  | 320 | 1.75819E-08 | 0 |
|  | 321 | 7.53509E-09 | 0.286610387 |
|  | 322 | 8.79094E-09 | 0.281515657 |
|  | 323 | 1.93401E-08 | 0.300109807 |
|  | 324 | 3.86801E-08 | 0.199783023 |
|  | 325 | 1.13762E-05 | 0.283587725 |
|  | 326 | 8.33884E-08 | 0.311502142 |
|  | 327 | 1.29913E-05 | 0.302759745 |
|  | 328 | 6.335E-06 | 0 |
|  | 329 | 4.39723E-06 | 0.309924551 |
|  | 330 | 1.33135E-05 | 0.095912486 |
|  | 331 | 3.04908E-05 | 0.283428483 |
|  | 332 | 1.65679E-05 | 0.19359384 |
|  | 333 | 1.31693E-05 | 0 |
|  | 334 | 0 | 0.249505638 |
|  | 335 | 0 | 0.110299238 |
|  | 336 | 0 | 0.267500514 |
|  | 337 | 0 | 0.11767417 |
|  | 338 | 0 | 0.100974727 |
|  | 339 | 0 | 0.082940414 |
|  | 340 | 6.78158E-09 | 0.180026174 |
|  | 341 | 1.36636E-07 | 0.300334593 |
|  | 342 | 3.89313E-08 | 0.283713119 |
|  | 343 | 5.14898E-08 | 0.257037406 |
|  | 344 | 6.42542E-06 | 0.192191007 |
|  | 345 | 3.33553E-06 | 0.278220837 |
|  | 346 | 4.01771E-06 | 0.277758537 |
|  | 347 | 1.15317E-05 | 0.153046032 |
|  | 348 | 2.06715E-05 | 0.159036963 |
|  | 349 | 1.98424E-05 | 0.108307256 |
|  | 350 | 9.43394E-06 | 0.249802983 |
|  | 351 | 8.59001E-07 | 0.104504338 |
|  | 352 | 2.95124E-07 | 0.377966061 |
|  | 353 | 2.56922E-06 | 0.125979599 |
|  | 354 | 2.03699E-07 | 0.153731277 |
|  | 355 | 3.72158E-06 | 0.087928131 |
|  | 356 | 1.56981E-07 | 0.320291376 |
|  | 357 | 2.00936E-07 | 0.100400759 |
|  | 358 | 0 | 0.386696166 |
|  | 359 | 5.90776E-06 | 0 |
|  | 360 | 1.18283E-05 | 0.144943491 |
|  | 361 | 7.36982E-06 | 0.278439095 |
|  | 362 | 7.86161E-06 | 0.064721698 |
|  | 363 | 9.66702E-06 | 0.084028371 |
|  | 364 | 5.02214E-06 | 0 |
|  | 365 | 3.4998E-06 | 0.250175502 |
|  | 366 | 4.12295E-06 | 0.218558947 |
|  | 367 | 2.73398E-06 | 0.312988185 |
|  | 368 | 5.81458E-07 | 0.361738299 |
|  | 369 | 1.18803E-07 | 0.126562141 |
|  | 370 | 0 | 0.202549852 |
|  | 371 | 0 | 0.37447211 |
|  | 372 | 0 | 0.417014271 |
|  | 373 | 0 | 0.411470882 |
|  | 374 | 0 | 0.391382033 |
|  | 375 | 0 | 0.377949171 |
|  | 376 | 0 | 0.366952348 |
|  | 377 | 0 | 0.335744447 |
|  | 378 | 0 | 0.243646748 |
|  | 379 | 0 | 0.286475518 |
|  | 380 | 0 | 0.240145955 |
|  | 381 | 0 | 0.32240181 |
|  | 382 | 0 | 0 |
|  | 383 | 0 | 0.243840659 |
|  | 384 | 0 | 0.214099969 |
|  | 385 | 1.10439E-06 | 0.258114387 |
|  | 386 | 8.53977E-09 | 0.261297255 |
|  | 387 | 1.10766E-07 | 0.289146986 |
|  | 388 | 9.18025E-07 | 0.240065855 |
|  | 389 | 0 | 0.254505791 |
|  | 390 | 0 | 0.260596768 |
|  | 391 | 0 | 0.147801855 |
|  | 392 | 0 | 0.059889119 |
|  | 393 | 0.004596337 | 0.006990783 |
|  | 394 | 6.01308E-05 | 0.018638157 |
|  | 395 | 0.000133909 | 0.028314403 |
|  | 396 | 0.000123815 | 0.036505225 |
|  | 397 | 3.1935E-05 | 0.050853586 |
|  | 398 | 4.12672E-07 | 0.068702294 |
|  | 399 | 3.07934E-07 | 0.167667282 |
|  | 400 | 3.7138E-06 | 0.142387745 |
|  | 401 | 1.15287E-07 | 0.209604554 |
|  | 402 | 2.40043E-06 | 0.220575214 |
|  | 403 | 1.40286E-05 | 0.032436101 |
|  | 404 | 4.03519E-05 | 0.056719897 |
|  | 405 | 4.4556E-05 | 0.050238599 |
|  | 406 | 0.00011183 | 0.046067117 |
|  | 407 | 4.05541E-05 | 0.047618716 |
|  | 408 | 0.000510755 | 0.084423183 |
|  | 409 | 6.26543E-06 | 0.114378823 |
|  | 410 | 1.17623E-06 | 0.157615888 |
|  | 411 | 7.13071E-07 | 0.125335465 |
|  | 412 | 1.22998E-06 | 0.255053789 |
|  | 413 | 1.61603E-06 | 0 |
|  | 414 | 2.97862E-06 | 0.167843365 |
|  | 415 | 0 | 0.165705168 |
|  | 416 | 0 | 0.126411506 |
|  | 417 | 0 | 0.18251267 |
|  | 418 | 9.47211E-06 | 0.098303409 |
| KWAI TSING | 419 | 0.000451252 | 0.043929557 |
|  | 420 | 6.63013E-06 | 0.222211062 |
|  | 421 | 8.3421E-06 | 0.195830199 |
|  | 422 | 3.99644E-05 | 0.149445333 |
|  | 423 | 7.90934E-07 | 0.153673536 |
|  | 424 | 1.73104E-05 | 0.204650081 |
|  | 425 | 1.6846E-05 | 0.168371563 |
|  | 426 | 2.49331E-05 | 0.165983274 |
|  | 427 | 1.01239E-05 | 0.15656479 |
|  | 428 | 5.36142E-05 | 0.188416681 |
|  | 429 | 0.000255258 | 0.15637053 |
|  | 430 | 4.77557E-05 | 0.225969319 |
|  | 431 | 1.35971E-05 | 0.196552771 |
|  | 432 | 9.09237E-05 | 0.166492393 |
|  | 433 | 6.59074E-05 | 0.176081251 |
|  | 434 | 0 | 0.175655687 |
|  | 435 | 0.000210031 | 0 |
|  | 436 | 0.000842665 | 0.071365884 |
|  | 437 | 9.72705E-06 | 0 |
|  | 438 | 1.0144E-05 | 0.091487538 |
|  | 439 | 1.89143E-05 | 0.075552279 |
|  | 440 | 5.50614E-06 | 0.115712654 |
|  | 441 | 2.00793E-05 | 0.087946079 |
|  | 442 | 2.28816E-07 | 0.142871103 |
|  | 443 | 1.43267E-06 | 0.138348519 |
|  | 444 | 2.34663E-05 | 0.101529848 |
|  | 445 | 0.007048047 | 0.035444627 |
|  | 446 | 0.000684275 | 0 |
|  | 447 | 0.000184333 | 0.128807375 |
|  | 448 | 0.000497035 | 0.092568374 |
|  | 449 | 0.00019371 | 0.070299729 |
|  | 450 | 4.68653E-05 | 0.082102998 |
|  | 451 | 6.02144E-05 | 0.069245079 |
|  | 452 | 0.002571243 | 0.060953699 |
|  | 453 | 0 | 0.064489874 |
|  | 454 | 0.003194895 | 0.015196641 |
|  | 455 | 2.81737E-06 | 0.061415642 |
|  | 456 | 0.000190918 | 0.081547282 |
|  | 457 | 1.23365E-05 | 0.075511173 |
|  | 458 | 0.000728861 | 0.082627645 |
|  | 459 | 0.000119287 | 0.091739991 |
|  | 460 | 1.90902E-05 | 0.031723248 |
|  | 461 | 5.56768E-06 | 0.091163727 |
|  | 462 | 5.29993E-06 | 0.044412183 |
|  | 463 | 4.6648E-05 | 0.083626701 |
|  | 464 | 4.30741E-05 | 0.094558505 |
|  | 465 | 6.75255E-05 | 0.10034846 |
|  | 466 | 3.32662E-05 | 0.089510229 |
|  | 467 | 0.000110368 | 0.082270088 |
|  | 468 | 6.60903E-06 | 0 |
|  | 469 | 5.07494E-05 | 0.091940117 |
|  | 470 | 6.7002E-06 | 0.082207471 |
|  | 471 | 4.89681E-06 | 0.087717752 |
|  | 472 | 8.55158E-06 | 0.078282467 |
|  | 473 | 1.09284E-05 | 0.062253244 |
|  | 474 | 1.21935E-05 | 0.072145768 |
|  | 475 | 0.003629393 | 0.021306712 |
|  | 476 | 3.06269E-05 | 0.058169847 |
|  | 477 | 0.007921008 | 0.007185329 |
| KWUN TONG | 478 | 9.59318E-06 | 0.327633147 |
|  | 479 | 1.08455E-06 | 0.316362586 |
|  | 480 | 0 | 0.276617915 |
|  | 481 | 2.85133E-05 | 0.127938674 |
|  | 482 | 0.000358291 | 0.197705516 |
|  | 483 | 7.50814E-05 | 0.243111458 |
|  | 484 | 2.55646E-05 | 0.18767645 |
|  | 485 | 0.001064655 | 0.056754098 |
|  | 486 | 3.43532E-05 | 0.155434055 |
|  | 487 | 5.45596E-05 | 0.129609391 |
|  | 488 | 0.000375602 | 0.409390682 |
|  | 489 | 3.94347E-05 | 0.255304952 |
|  | 490 | 1.93431E-05 | 0.186282116 |
|  | 491 | 1.26364E-05 | 0.130252446 |
|  | 492 | 4.40275E-06 | 0.193254847 |
|  | 493 | 5.93288E-06 | 0.329585134 |
|  | 494 | 0.000471882 | 0.12005954 |
|  | 495 | 1.38143E-08 | 0.111136873 |
|  | 496 | 6.29431E-07 | 0.078972561 |
|  | 497 | 0.000352088 | 0.070153392 |
|  | 498 | 5.99444E-05 | 0.094156538 |
|  | 499 | 3.45931E-05 | 0.196360867 |
|  | 500 | 2.8172E-05 | 0.19897508 |
|  | 501 | 1.82686E-05 | 0.198538864 |
|  | 502 | 0 | 0.199637151 |
|  | 503 | 3.30288E-06 | 0.18648442 |
|  | 504 | 0.000313296 | 0.232915895 |
|  | 505 | 1.70338E-05 | 0.238469765 |
|  | 506 | 1.96575E-05 | 0.274076242 |
|  | 507 | 1.63846E-05 | 0.187574593 |
|  | 508 | 6.52514E-06 | 0.230900863 |
|  | 509 | 9.9639E-06 | 0.246697783 |
|  | 510 | 2.50039E-06 | 0.295184532 |
|  | 511 | 1.80071E-05 | 0.290901217 |
|  | 512 | 1.74608E-05 | 0.295384309 |
|  | 513 | 1.12273E-06 | 0.283534316 |
|  | 514 | 0 | 0.266935743 |
|  | 515 | 0.000160211 | 0.215775695 |
|  | 516 | 5.16882E-06 | 0.221029811 |
|  | 517 | 5.26301E-06 | 0.13829515 |
|  | 518 | 5.26402E-06 | 0.099031144 |
|  | 519 | 1.01355E-05 | 0.078086893 |
|  | 520 | 1.18027E-05 | 0 |
|  | 521 | 0 | 0.300840934 |
|  | 522 | 5.45139E-06 | 0.319545875 |
|  | 523 | 5.97684E-06 | 0.16270104 |
|  | 524 | 4.40552E-06 | 0.153686562 |
|  | 525 | 4.77574E-06 | 0.325724141 |
|  | 526 | 7.62792E-05 | 0.294094993 |
|  | 527 | 3.06477E-06 | 0.2446083 |
|  | 528 | 1.84459E-06 | 0.199454226 |
|  | 529 | 7.26282E-06 | 0.192081333 |
|  | 530 | 4.53693E-05 | 0.192867939 |
|  | 531 | 7.60238E-05 | 0.162237803 |
|  | 532 | 1.77871E-05 | 0.230197765 |
|  | 533 | 2.59473E-05 | 0.243611353 |
|  | 534 | 2.27E-05 | 0.169657366 |
|  | 535 | 1.15458E-05 | 0.240796833 |
|  | 536 | 8.58157E-05 | 0.218215673 |
|  | 537 | 4.57124E-05 | 0.246909775 |
|  | 538 | 4.52505E-05 | 0.234730114 |
|  | 539 | 3.98792E-05 | 0.212376018 |
|  | 540 | 2.49555E-05 | 0.218591492 |
|  | 541 | 4.06523E-05 | 0.215326442 |
|  | 542 | 0.000195609 | 0.233355896 |
|  | 543 | 0.000162845 | 0.164760596 |
|  | 544 | 2.6204E-05 | 0.164726551 |
|  | 545 | 0.000286932 | 0.098603025 |
|  | 546 | 0.00028518 | 0.113591247 |
|  | 547 | 3.19533E-05 | 0.238135482 |
|  | 548 | 6.63251E-05 | 0.236689667 |
|  | 549 | 2.3073E-05 | 0.169321263 |
|  | 550 | 0.000108123 | 0.187121302 |
|  | 551 | 0.000168728 | 0.13022768 |
|  | 552 | 1.35438E-05 | 0.168346071 |
|  | 553 | 5.58602E-07 | 0.140479613 |
|  | 554 | 0.000398387 | 0.158649173 |
|  | 555 | 0.000355266 | 0.138359981 |
|  | 556 | 4.25198E-05 | 0.139239586 |
|  | 557 | 5.58775E-05 | 0.192590461 |
| NORTH | 558 | 0.010171849 | 0.00099782 |
|  | 559 | 0.001168673 | 0.002711926 |
|  | 560 | 0.000945128 | 0.003706448 |
|  | 561 | 0.051127607 | 0.000808234 |
|  | 562 | 0.024836326 | 7.5404E-05 |
|  | 563 | 0.0532551 | 1.79141E-07 |
|  | 564 | 0.0053711 | 0.003928708 |
|  | 565 | 0.001125648 | 0.007545542 |
|  | 566 | 0.053603609 | 0.0016537 |
|  | 567 | 0.07200929 | 0.000615663 |
|  | 568 | 0.004722815 | 0.008243207 |
|  | 569 | 0.000395492 | 0.007320155 |
|  | 570 | 0.002379278 | 0.007367423 |
|  | 571 | 4.09407E-07 | 0.029009478 |
|  | 572 | 0.000636304 | 0.03561262 |
|  | 573 | 0.000141102 | 0.06668224 |
|  | 574 | 0.000211876 | 0.084651587 |
|  | 575 | 1.54876E-05 | 0.075320921 |
|  | 576 | 3.48624E-06 | 0.092223023 |
|  | 577 | 1.66126E-05 | 0.079959663 |
|  | 578 | 5.34459E-05 | 0.053377455 |
|  | 579 | 3.52429E-05 | 0.068937779 |
|  | 580 | 0.00096092 | 0.014158985 |
|  | 581 | 0.001591929 | 0.046686617 |
|  | 582 | 0.000106572 | 0.041192047 |
|  | 583 | 0.002142346 | 0.038474215 |
|  | 584 | 0.002142346 | 0.014612801 |
|  | 585 | 7.86837E-05 | 0.030889557 |
|  | 586 | 2.51858E-05 | 0.044536042 |
|  | 587 | 0.000121439 | 0.042533209 |
|  | 588 | 0.000236763 | 0.020329284 |
|  | 589 | 0.000355341 | 0.028913332 |
|  | 590 | 0.000166059 | 0.039701259 |
|  | 591 | 6.99744E-05 | 0.028717153 |
|  | 592 | 4.43006E-05 | 0.042271966 |
|  | 593 | 0.000117061 | 0.037890907 |
|  | 594 | 4.78611E-05 | 0.03245528 |
|  | 595 | 1.54653E-05 | 0.021485377 |
|  | 596 | 0.000109611 | 0.039536321 |
|  | 597 | 3.88303E-05 | 0.04141215 |
|  | 598 | 0.000230458 | 0.040561736 |
|  | 599 | 0.000828076 | 0.034867714 |
|  | 600 | 0.001210669 | 0.019237329 |
|  | 601 | 3.77697E-05 | 0.076312351 |
|  | 602 | 8.93863E-06 | 0.078508462 |
|  | 603 | 7.06164E-05 | 0.062052389 |
|  | 604 | 0.000101736 | 0.044805405 |
|  | 605 | 5.749E-05 | 0.067056164 |
|  | 606 | 8.83389E-05 | 0.071612059 |
|  | 607 | 1.5128E-06 | 0.033450391 |
|  | 608 | 2.82689E-05 | 0.040178545 |
|  | 609 | 0.000470906 | 0.031498195 |
|  | 610 | 0.000180972 | 0.020147222 |
|  | 611 | 4.65953E-05 | 0.047302014 |
|  | 612 | 1.75158E-05 | 0.057034273 |
|  | 613 | 4.36031E-07 | 0.059218789 |
|  | 614 | 8.62015E-06 | 0.052677454 |
|  | 615 | 1.83203E-06 | 0.051018423 |
|  | 616 | 2.47704E-06 | 0.039327817 |
|  | 617 | 0.002287082 | 0.024909832 |
|  | 618 | 0.000281017 | 0.020452604 |
|  | 619 | 1.15819E-05 | 0.06214716 |
|  | 620 | 0.00084695 | 0.015594989 |
|  | 621 | 0.058111473 | 0.00519393 |
|  | 622 | 0.000294463 | 0.0146576 |
|  | 623 | 0.004785162 | 0.007682174 |
|  | 624 | 0.001676084 | 0.002993823 |
|  | 625 | 0.051690439 | 0.001969651 |
|  | 626 | 0.001313067 | 0.00118477 |
|  | 627 | 0.011695998 | 0.004077039 |
|  | 628 | 0.001968841 | 0.001397345 |
|  | 629 | 0.000299735 | 0.00467576 |
|  | 630 | 0.011345515 | 0.001141284 |
|  | 631 | 0.036001615 | 0.00020054 |
|  | 632 | 0.026977147 | 0.000417202 |
|  | 633 | 0.101043392 | 0.000211277 |
|  | 634 | 0.000399267 | 0 |
|  | 635 | 0.355169639 | 0 |
|  | 636 | 0.000127661 | 0 |
| SAI KUNG | 637 | 0.000628812 | 0.099505515 |
|  | 638 | 0.073591671 | 0.000205154 |
|  | 639 | 1 | 2.33381E-05 |
|  | 640 | 0.015094491 | 0.003103973 |
|  | 641 | 0.026885748 | 0.002550352 |
|  | 642 | 0.00035372 | 0.002791635 |
|  | 643 | 0.159039436 | 0.000961895 |
|  | 644 | 0.02360773 | 0.001432624 |
|  | 645 | 0.019676195 | 0.005120939 |
|  | 646 | 0.000487144 | 0.01740151 |
|  | 647 | 0.005941042 | 0.007089001 |
|  | 648 | 0.025262361 | 0.000794908 |
|  | 649 | 0.015390099 | 0.000187998 |
|  | 650 | 0.001269128 | 0.002174299 |
|  | 651 | 0.000557343 | 0.028081883 |
|  | 652 | 0.004689598 | 0.013505844 |
|  | 653 | 7.29718E-05 | 0.027608529 |
|  | 654 | 0.000448462 | 0.030712395 |
|  | 655 | 0.001047614 | 0.015992873 |
|  | 656 | 0.016971727 | 0.002942647 |
|  | 657 | 0.016964402 | 0.001459157 |
|  | 658 | 0.010170301 | 0.002971501 |
|  | 659 | 0.009333218 | 0.004668066 |
|  | 660 | 0.001908473 | 0.002987364 |
|  | 661 | 0.010793113 | 0.006240334 |
|  | 662 | 0.012801898 | 0.003579533 |
|  | 663 | 0.002590305 | 0.010554678 |
|  | 664 | 0.012592835 | 0.013448312 |
|  | 665 | 0.001101333 | 0.033929205 |
|  | 666 | 0.001436437 | 0.006229307 |
|  | 667 | 0.022608808 | 0.003119367 |
|  | 668 | 0.00328816 | 0.004806312 |
|  | 669 | 0.125400258 | 6.89419E-05 |
|  | 670 | 0.006722939 | 0.005540644 |
|  | 671 | 0.000159364 | 0.094849228 |
|  | 672 | 0.001510901 | 0.048590458 |
|  | 673 | 0.000637469 | 0.07175399 |
|  | 674 | 1.52184E-06 | 0.110819179 |
|  | 675 | 4.015E-05 | 0.043727035 |
|  | 676 | 0.005737984 | 0.048532042 |
|  | 677 | 0.012699678 | 0.030015104 |
|  | 678 | 0.000108888 | 0.074439796 |
|  | 679 | 0.001331229 | 0.081915314 |
|  | 680 | 2.80069E-05 | 0.100667204 |
|  | 681 | 8.7613E-06 | 0.102098523 |
|  | 682 | 6.12352E-07 | 0.104884725 |
|  | 683 | 6.27924E-07 | 0.095342248 |
|  | 684 | 0.000588359 | 0.061566613 |
|  | 685 | 5.88466E-06 | 0.104328479 |
|  | 686 | 0.000302294 | 0.116182165 |
|  | 687 | 0.003375495 | 0.06666158 |
|  | 688 | 9.0818E-06 | 0.112428998 |
|  | 689 | 0.000138832 | 0.143456979 |
|  | 690 | 6.13784E-06 | 0.155195789 |
|  | 691 | 1.50647E-05 | 0.134463953 |
|  | 692 | 2.09719E-05 | 0.158641501 |
|  | 693 | 2.717E-05 | 0.110301765 |
|  | 694 | 4.92556E-05 | 0.166221099 |
|  | 695 | 6.86799E-06 | 0.162813414 |
|  | 696 | 0.000151691 | 0.123188966 |
|  | 697 | 4.17394E-06 | 0.161783408 |
|  | 698 | 6.52891E-06 | 0.161599171 |
|  | 699 | 7.12493E-06 | 0.195678926 |
|  | 700 | 1.29448E-05 | 0.207082502 |
|  | 701 | 7.73653E-06 | 0.192021243 |
|  | 702 | 9.83757E-06 | 0.106510841 |
|  | 703 | 7.01768E-06 | 0.130521207 |
|  | 704 | 0.010530437 | 0.023994731 |
|  | 705 | 0.000889392 | 0.012487696 |
| SHA TIN | 706 | 0.127201875 | 0.034756012 |
|  | 707 | 0.025230358 | 0.011785965 |
|  | 708 | 0.014647546 | 0.026291193 |
|  | 709 | 0.003512394 | 0.005940311 |
|  | 710 | 3.34056E-07 | 0.056913058 |
|  | 711 | 6.54146E-06 | 0.03468809 |
|  | 712 | 0.002311976 | 0.026498746 |
|  | 713 | 0.003975753 | 0.003339244 |
|  | 714 | 0.000503102 | 0.034642367 |
|  | 715 | 0.016202171 | 0.012991519 |
|  | 716 | 0.00010557 | 0.055142425 |
|  | 717 | 4.78471E-05 | 0.145865854 |
|  | 718 | 0.000110629 | 0.099651877 |
|  | 719 | 5.19401E-05 | 0.159533617 |
|  | 720 | 1.7813E-05 | 0.152444143 |
|  | 721 | 6.52745E-05 | 0.168402329 |
|  | 722 | 7.76423E-05 | 0.157389718 |
|  | 723 | 3.27493E-05 | 0.107138204 |
|  | 724 | 9.0927E-05 | 0.135850987 |
|  | 725 | 6.24189E-05 | 0.108663053 |
|  | 726 | 0.000973852 | 0.085902214 |
|  | 727 | 6.1354E-05 | 0 |
|  | 728 | 0.000210557 | 0.045323383 |
|  | 729 | 0.020603339 | 0.039594554 |
|  | 730 | 0.0004288 | 0.070086545 |
|  | 731 | 0.000151445 | 0.083393066 |
|  | 732 | 1.71438E-05 | 0.07436236 |
|  | 733 | 1.11409E-05 | 0.11023764 |
|  | 734 | 0.000290639 | 0.056004457 |
|  | 735 | 1.70183E-05 | 0.104679485 |
|  | 736 | 3.54149E-08 | 0.115086085 |
|  | 737 | 7.08299E-08 | 0.087406662 |
|  | 738 | 0.000124814 | 0.053305022 |
|  | 739 | 0.000266548 | 0.069004093 |
|  | 740 | 0.000358756 | 0.050499892 |
|  | 741 | 0.000292121 | 0.068186739 |
|  | 742 | 0.000479475 | 0.040005416 |
|  | 743 | 5.83952E-05 | 0.062252009 |
|  | 744 | 7.93666E-05 | 0.039374503 |
|  | 745 | 0.005561068 | 0.027877482 |
|  | 746 | 0.007264487 | 0.031353785 |
|  | 747 | 0.682043105 | 0.006524934 |
|  | 748 | 4.26552E-05 | 0.079955803 |
|  | 749 | 0.000447298 | 0.069914213 |
|  | 750 | 9.48543E-06 | 0.076223373 |
|  | 751 | 4.45716E-05 | 0.028477824 |
|  | 752 | 8.2023E-05 | 0.018915685 |
|  | 753 | 8.2023E-05 | 0.018548389 |
|  | 754 | 8.26688E-05 | 0.01847838 |
|  | 755 | 0.005368481 | 0.009449565 |
|  | 756 | 0.005365922 | 0.008946854 |
|  | 757 | 0 | 0.05018965 |
|  | 758 | 0.000150394 | 0.032586687 |
|  | 759 | 0 | 0.074872713 |
|  | 760 | 0 | 0.074161921 |
|  | 761 | 0 | 0.09081689 |
|  | 762 | 0 | 0.095851878 |
|  | 763 | 0 | 0.13046094 |
|  | 764 | 3.36367E-06 | 0.108132456 |
|  | 765 | 0 | 0.127577983 |
|  | 766 | 0 | 0.109531711 |
|  | 767 | 0 | 0.122594738 |
|  | 768 | 0 | 0.088333535 |
|  | 769 | 0 | 0.100370477 |
|  | 770 | 0 | 0.109161645 |
|  | 771 | 0 | 0.115001483 |
|  | 772 | 0 | 0.113639861 |
|  | 773 | 0 | 0.102148028 |
|  | 774 | 0.000966251 | 0.039668056 |
|  | 775 | 0.000253118 | 0.077703093 |
|  | 776 | 0.000131579 | 0.087187209 |
|  | 777 | 0.00025547 | 0.080739078 |
|  | 778 | 0.000106785 | 0.040538117 |
|  | 779 | 0.000382448 | 0.019775541 |
|  | 780 | 0.000601512 | 0.006550545 |
|  | 781 | 0.000235427 | 0.081397028 |
|  | 782 | 0.000346095 | 0.142595422 |
|  | 783 | 4.71988E-05 | 0.155314154 |
|  | 784 | 5.11429E-05 | 0.155857832 |
|  | 785 | 0 | 0.137317254 |
|  | 786 | 0.000163245 | 0.081555738 |
|  | 787 | 0.00551753 | 0.067996173 |
|  | 788 | 3.62219E-05 | 0.125250366 |
|  | 789 | 0.009138546 | 0.043515219 |
|  | 790 | 7.36091E-05 | 0.090976368 |
|  | 791 | 8.70615E-05 | 0.062863913 |
|  | 792 | 9.15004E-05 | 0.075584693 |
|  | 793 | 0.000215691 | 0.094456155 |
|  | 794 | 0.000128228 | 0.139541668 |
|  | 795 | 8.03743E-09 | 0.146107913 |
|  | 796 | 2.33965E-06 | 0.171037422 |
|  | 797 | 2.73122E-06 | 0.111662762 |
|  | 798 | 9.51833E-06 | 0.163947168 |
|  | 799 | 7.55589E-05 | 0.139615184 |
|  | 800 | 5.54784E-06 | 0.114825563 |
|  | 801 | 0.000669402 | 0.066946363 |
|  | 802 | 0.000633479 | 0.057222822 |
|  | 803 | 0.000256325 | 0.101485123 |
|  | 804 | 0.00059162 | 0.041045284 |
|  | 805 | 1.98952E-06 | 0.065106305 |
|  | 806 | 0.000615976 | 0.104356842 |
|  | 807 | 6.98855E-05 | 0 |
|  | 808 | 2.79326E-06 | 0.121566467 |
|  | 809 | 3.40059E-06 | 0.075015553 |
|  | 810 | 1.18452E-06 | 0.074524373 |
| SHAM SHUI PO | 811 | 3.70174E-06 | 0.147200811 |
|  | 812 | 1.34095E-05 | 0.122907657 |
|  | 813 | 3.90494E-06 | 0.253469212 |
|  | 814 | 4.00003E-05 | 0.067532732 |
|  | 815 | 6.14715E-05 | 0.095096978 |
|  | 816 | 0.00013185 | 0.114744029 |
|  | 817 | 2.10091E-05 | 0.094505427 |
|  | 818 | 1.88209E-05 | 0.075629304 |
|  | 819 | 0.000106266 | 0.093284382 |
|  | 820 | 6.70776E-05 | 0.058215734 |
|  | 821 | 0.00481769 | 0.097412438 |
|  | 822 | 0.000156113 | 0.132337096 |
|  | 823 | 0.000395125 | 0 |
|  | 824 | 2.71263E-08 | 0.223991553 |
|  | 825 | 0.000140023 | 0.106384251 |
|  | 826 | 0.000390163 | 0.203034758 |
|  | 827 | 9.97001E-05 | 0.208306284 |
|  | 828 | 0.00020527 | 0.197051686 |
|  | 829 | 2.18377E-05 | 0.46371447 |
|  | 830 | 1.44754E-05 | 0.303314534 |
|  | 831 | 1.80378E-05 | 0.527201689 |
|  | 832 | 4.01972E-05 | 0.338961136 |
|  | 833 | 9.2166E-05 | 0.262239075 |
|  | 834 | 4.93275E-05 | 0.445227757 |
|  | 835 | 4.34059E-05 | 0.338315801 |
|  | 836 | 1.91155E-05 | 0.250281324 |
|  | 837 | 4.11883E-05 | 0.168047978 |
|  | 838 | 0.000719221 | 0.019118006 |
|  | 839 | 0.004581913 | 0.09304005 |
|  | 840 | 3.8962E-05 | 0.147143256 |
|  | 841 | 0 | 0.240578774 |
|  | 842 | 0.000214045 | 0.233097388 |
|  | 843 | 6.10342E-08 | 0.277719053 |
|  | 844 | 0 | 0.105312111 |
|  | 845 | 0 | 0.281491289 |
|  | 846 | 0 | 0.153142585 |
|  | 847 | 1.91643E-07 | 0.341658864 |
|  | 848 | 2.22109E-06 | 0.21981291 |
|  | 849 | 0 | 0.362446803 |
|  | 850 | 2.91106E-07 | 0.403525554 |
|  | 851 | 3.61433E-07 | 0 |
|  | 852 | 1.0524E-06 | 0.467215451 |
|  | 853 | 1.02779E-06 | 0.207133485 |
|  | 854 | 1.00149E-05 | 0.533763339 |
|  | 855 | 1.70514E-05 | 0 |
|  | 856 | 2.33259E-05 | 0.307306897 |
|  | 857 | 7.36103E-06 | 0.601334322 |
|  | 858 | 4.30839E-05 | 0.542626512 |
|  | 859 | 1.72481E-05 | 0.613673948 |
|  | 860 | 0 | 0.292107535 |
|  | 861 | 6.50278E-07 | 0.585971993 |
|  | 862 | 1.06998E-06 | 0.64737385 |
|  | 863 | 9.98651E-07 | 0.453742162 |
|  | 864 | 1.06998E-07 | 0.605916702 |
|  | 865 | 4.85511E-07 | 0.496367607 |
|  | 866 | 1.68334E-06 | 0.309341017 |
|  | 867 | 6.12352E-07 | 0.382160872 |
|  | 868 | 2.70761E-07 | 0.549055579 |
|  | 869 | 1.54469E-07 | 0.580774056 |
|  | 870 | 1.10515E-08 | 0.6406563 |
|  | 871 | 5.60109E-08 | 0.658407561 |
|  | 872 | 8.41419E-08 | 0.697947729 |
|  | 873 | 2.63728E-07 | 0.697363503 |
|  | 874 | 6.05821E-07 | 0.712747184 |
|  | 875 | 7.95203E-07 | 0.690778394 |
|  | 876 | 1.36862E-06 | 0.682394254 |
|  | 877 | 6.06851E-06 | 0 |
|  | 878 | 1.23671E-05 | 0.64080435 |
|  | 879 | 2.63728E-08 | 0.317524717 |
|  | 880 | 1.37892E-07 | 0.342322808 |
|  | 881 | 2.70761E-07 | 0.711431634 |
|  | 882 | 3.09943E-07 | 0.718939877 |
|  | 883 | 5.00581E-07 | 0 |
|  | 884 | 8.83866E-07 | 0.70898887 |
|  | 885 | 1.15639E-06 | 0 |
|  | 886 | 1.62004E-06 | 0.655511784 |
|  | 887 | 6.77229E-06 | 0.305457533 |
|  | 888 | 1.93652E-07 | 0.622305952 |
|  | 889 | 2.39365E-07 | 0 |
|  | 890 | 3.62187E-07 | 0.345435105 |
|  | 891 | 5.40266E-07 | 0.359534659 |
|  | 892 | 1.05742E-06 | 0.721196283 |
|  | 893 | 1.05542E-06 | 0.703808672 |
|  | 894 | 1.24053E-06 | 0.686743201 |
|  | 895 | 1.34727E-06 | 0.646156523 |
|  | 896 | 9.90613E-07 | 0 |
|  | 897 | 1.37666E-06 | 0.582540164 |
|  | 898 | 4.66673E-07 | 0.480616181 |
|  | 899 | 2.62975E-07 | 0 |
|  | 900 | 4.17444E-07 | 0.338620388 |
|  | 901 | 5.42024E-06 | 0.70138087 |
|  | 902 | 8.2356E-06 | 0.707216355 |
|  | 903 | 6.51534E-06 | 0.676210217 |
|  | 904 | 6.44677E-06 | 0.667369043 |
|  | 905 | 1.10866E-06 | 0.147200749 |
|  | 906 | 5.27708E-07 | 0.418688659 |
|  | 907 | 5.75982E-06 | 0.339081614 |
|  | 908 | 1.85504E-05 | 0.336799749 |
|  | 909 | 1.4439E-05 | 0 |
|  | 910 | 1.99801E-05 | 0.62700853 |
|  | 911 | 2.59456E-05 | 0.588644381 |
|  | 912 | 1.84663E-05 | 0.532534816 |
|  | 913 | 7.20104E-07 | 0.651426333 |
|  | 914 | 1.98075E-05 | 0.41686868 |
|  | 915 | 4.30995E-05 | 0.631293132 |
|  | 916 | 6.8707E-05 | 0.286985617 |
|  | 917 | 3.11943E-05 | 0.580038098 |
|  | 918 | 5.98517E-05 | 0 |
|  | 919 | 0.000103599 | 0.49782735 |
|  | 920 | 5.25317E-05 | 0.352433714 |
|  | 921 | 0.000920032 | 0.077350197 |
|  | 922 | 8.58147E-06 | 0.178555425 |
|  | 923 | 3.6493E-05 | 0.087222403 |
|  | 924 | 0.000189895 | 0.305453321 |
|  | 925 | 1.71371E-05 | 0.104287377 |
|  | 926 | 0 | 0.084984833 |
|  | 927 | 8.19607E-05 | 0.157461641 |
|  | 928 | 2.74541E-05 | 0.111226434 |
|  | 929 | 1.31101E-05 | 0.067916281 |
|  | 930 | 0.000791341 | 0.070875421 |
| SOUTHERN | 931 | 0.007886717 | 0.012231207 |
|  | 932 | 0.002120094 | 0.007064051 |
|  | 933 | 0.002961848 | 0.00958258 |
|  | 934 | 0.002337681 | 0.016839631 |
|  | 935 | 8.32728E-06 | 0.022402464 |
|  | 936 | 0.001124468 | 0.038508142 |
|  | 937 | 0.002701069 | 0.016621238 |
|  | 938 | 0.000766741 | 0.034743536 |
|  | 939 | 0.00037638 | 0.050559483 |
|  | 940 | 2.13926E-05 | 0.042414404 |
|  | 941 | 9.16518E-07 | 0.049482228 |
|  | 942 | 0.000208097 | 0.022543054 |
|  | 943 | 8.64752E-05 | 0.013055005 |
|  | 944 | 0.00084119 | 0.053301836 |
|  | 945 | 3.88886E-06 | 0.062369127 |
|  | 946 | 0.0061806 | 0.048602855 |
|  | 947 | 1.02806E-05 | 0.062249505 |
|  | 948 | 1.22101E-05 | 0.038154247 |
|  | 949 | 1.49722E-05 | 0.062023256 |
|  | 950 | 3.70048E-06 | 0.053242984 |
|  | 951 | 0.000173144 | 0.076382021 |
|  | 952 | 3.34483E-06 | 0.021692278 |
|  | 953 | 0 | 0.063934944 |
|  | 954 | 0.000199648 | 0.019056149 |
|  | 955 | 8.28725E-05 | 0.049515229 |
|  | 956 | 0.000895803 | 0.022853067 |
|  | 957 | 0.000475857 | 0.054314459 |
|  | 958 | 0.000558705 | 0.045432328 |
|  | 959 | 4.77373E-06 | 0.038301255 |
|  | 960 | 1.68055E-05 | 0.055629831 |
|  | 961 | 2.98463E-05 | 0.050237668 |
|  | 962 | 6.441E-06 | 0.065477781 |
|  | 963 | 2.64874E-05 | 0.063686683 |
|  | 964 | 7.15432E-06 | 0.068259962 |
|  | 965 | 0.000119539 | 0.028617149 |
|  | 966 | 3.21573E-05 | 0.017298334 |
|  | 967 | 0.000145277 | 0.057501146 |
|  | 968 | 0.006189414 | 0.059476291 |
|  | 969 | 0.013435465 | 0.022064383 |
|  | 970 | 0.000196391 | 0.043429631 |
|  | 971 | 0.000196188 | 0.053729385 |
|  | 972 | 0.002635194 | 0.007566817 |
|  | 973 | 0.014718463 | 0.005018866 |
|  | 974 | 0.000246284 | 0.011135511 |
|  | 975 | 0.009173394 | 0.010387947 |
|  | 976 | 0.001557596 | 0.005908031 |
|  | 977 | 0.010190116 | 0.000878022 |
|  | 978 | 0.050605051 | 0.004763015 |
|  | 979 | 0.007980679 | 0.003135444 |
|  | 980 | 0.002062458 | 0.009571361 |
|  | 981 | 0.00033507 | 0.006719861 |
|  | 982 | 0.002259408 | 0.001733882 |
|  | 983 | 0.001002848 | 0.006182115 |
|  | 984 | 0.072957209 | 0.004632654 |
|  | 985 | 0.001946295 | 0.006443082 |
|  | 986 | 0.000548607 | 0.003543164 |
|  | 987 | 0.048863614 | 0.000187658 |
| TAI PO | 988 | 0.013349612 | 0.004007405 |
|  | 989 | 0.001137513 | 0.005112944 |
|  | 990 | 0.101436773 | 0.000667992 |
|  | 991 | 1.21112E-05 | 0.004223695 |
|  | 992 | 0.393809341 | 0.001493349 |
|  | 993 | 0.005972405 | 0.011325291 |
|  | 994 | 0.012547468 | 0.009513661 |
|  | 995 | 0.000164433 | 0.077726393 |
|  | 996 | 0.00106251 | 0.037570495 |
|  | 997 | 0.000122371 | 0 |
|  | 998 | 0.011599618 | 0.042581254 |
|  | 999 | 4.16314E-06 | 0.096405529 |
|  | 1000 | 0.00013821 | 0.002436795 |
|  | 1001 | 0.006024121 | 0.006451272 |
|  | 1002 | 0.00226599 | 0.007149913 |
|  | 1003 | 1.20991E-05 | 0.093465335 |
|  | 1004 | 1.81184E-05 | 0.084823085 |
|  | 1005 | 3.6118E-05 | 0.107527046 |
|  | 1006 | 2.27324E-05 | 0.083888781 |
|  | 1007 | 2.4476E-05 | 0.146143943 |
|  | 1008 | 1.46191E-05 | 0.13752462 |
|  | 1009 | 6.33026E-05 | 0.0474138 |
|  | 1010 | 0.000110902 | 0.130028895 |
|  | 1011 | 4.3315E-05 | 0.177156744 |
|  | 1012 | 8.41537E-05 | 0.148724265 |
|  | 1013 | 8.50323E-05 | 0.050889154 |
|  | 1014 | 0.000253039 | 0.104002178 |
|  | 1015 | 0.003288691 | 0.056973979 |
|  | 1016 | 2.64572E-05 | 0.080396899 |
|  | 1017 | 0.001692166 | 0.032439192 |
|  | 1018 | 5.40861E-05 | 0.019529473 |
|  | 1019 | 6.85796E-05 | 0.054424906 |
|  | 1020 | 0.000100713 | 0.098143377 |
|  | 1021 | 3.29027E-05 | 0.050131344 |
|  | 1022 | 3.61393E-05 | 0.079178783 |
|  | 1023 | 0.007838771 | 0.001554552 |
|  | 1024 | 0.015605125 | 0.00153923 |
|  | 1025 | 0.118484718 | 0.001567889 |
|  | 1026 | 0.025843326 | 0.004942689 |
|  | 1027 | 0.011127553 | 0.010141037 |
|  | 1028 | 3.14763E-05 | 0.097395963 |
|  | 1029 | 1.14486E-05 | 0.154506511 |
|  | 1030 | 1.99037E-05 | 0.15875567 |
|  | 1031 | 0.000447907 | 0.138923285 |
|  | 1032 | 4.82035E-05 | 0.118068636 |
|  | 1033 | 5.69477E-06 | 0.17903662 |
|  | 1034 | 2.00649E-05 | 0.162925099 |
|  | 1035 | 1.24025E-05 | 0.171234407 |
|  | 1036 | 3.32315E-05 | 0.158024062 |
|  | 1037 | 2.05339E-05 | 0.149862667 |
|  | 1038 | 2.61571E-05 | 0.138220508 |
|  | 1039 | 2.98138E-07 | 0.164531137 |
|  | 1040 | 0.000102683 | 0.13034341 |
|  | 1041 | 3.94542E-05 | 0.124911425 |
|  | 1042 | 2.11927E-05 | 0.110940169 |
|  | 1043 | 4.97909E-05 | 0.08796846 |
|  | 1044 | 1.81013E-05 | 0.075010883 |
|  | 1045 | 4.74208E-06 | 0.127685681 |
|  | 1046 | 3.60725E-05 | 0 |
|  | 1047 | 8.91904E-07 | 0.135702898 |
|  | 1048 | 0.000597492 | 0.006928987 |
|  | 1049 | 0.005270415 | 0.00644977 |
|  | 1050 | 0.005523714 | 0.002529346 |
|  | 1051 | 0.005003221 | 0.005444674 |
|  | 1052 | 0.000721322 | 0.002450959 |
|  | 1053 | 0.009830555 | 0.007412576 |
|  | 1054 | 0.013557109 | 0.002874613 |
|  | 1055 | 0.001683875 | 0.007778013 |
|  | 1056 | 0.003626904 | 0.007424931 |
|  | 1057 | 0.005096603 | 0.003031418 |
|  | 1058 | 0.001223429 | 0.009064921 |
|  | 1059 | 0.002062289 | 0.015949289 |
|  | 1060 | 0.000102318 | 0.011334446 |
|  | 1061 | 0.016937227 | 0.000990459 |
|  | 1062 | 0.000504422 | 0.000639341 |
|  | 1063 | 0.573828717 | 0.000116358 |
|  | 1064 | 0.000648642 | 0.002727728 |
|  | 1065 | 0.005444283 | 0.003229723 |
|  | 1066 | 0.001178404 | 0.002714122 |
| TSUEN WAN | 1067 | 0.388108593 | 0.001525409 |
|  | 1068 | 0.002865695 | 0.02737486 |
|  | 1069 | 0.000874104 | 0.030495584 |
|  | 1070 | 0.007118124 | 0.021693813 |
|  | 1071 | 0.000235713 | 0.096027004 |
|  | 1072 | 0.002387454 | 0.044649842 |
|  | 1073 | 0.000125285 | 0.082919578 |
|  | 1074 | 4.8197E-06 | 0.171894434 |
|  | 1075 | 0.000770764 | 0.032492992 |
|  | 1076 | 4.44395E-05 | 0.055741716 |
|  | 1077 | 0.000552521 | 0.045983421 |
|  | 1078 | 8.73232E-05 | 0.041536077 |
|  | 1079 | 1.02869E-05 | 0.055536967 |
|  | 1080 | 4.9823E-05 | 0.040606332 |
|  | 1081 | 0.00015426 | 0.028540124 |
|  | 1082 | 5.11271E-05 | 0.026826046 |
|  | 1083 | 0.000850041 | 0.012586458 |
|  | 1084 | 2.71062E-05 | 0.162801346 |
|  | 1085 | 2.07692E-06 | 0.280868018 |
|  | 1086 | 6.54548E-07 | 0.210192121 |
|  | 1087 | 6.48771E-07 | 0.266665352 |
|  | 1088 | 7.89577E-06 | 0.293402277 |
|  | 1089 | 2.08124E-05 | 0.287546429 |
|  | 1090 | 2.23742E-06 | 0.323508692 |
|  | 1091 | 7.80183E-06 | 0.23724067 |
|  | 1092 | 1.14031E-07 | 0.231685482 |
|  | 1093 | 0 | 0.31028609 |
|  | 1094 | 4.75512E-05 | 0.228468981 |
|  | 1095 | 4.80211E-06 | 0.158589256 |
|  | 1096 | 3.65527E-06 | 0.245068206 |
|  | 1097 | 2.21313E-05 | 0.271788626 |
|  | 1098 | 8.30659E-05 | 0.208906565 |
|  | 1099 | 1.34715E-05 | 0.279805528 |
|  | 1100 | 9.071E-06 | 0.310410656 |
|  | 1101 | 5.04851E-08 | 0.305070741 |
|  | 1102 | 2.16031E-06 | 0.295985759 |
|  | 1103 | 8.73292E-06 | 0.082972622 |
|  | 1104 | 7.09203E-06 | 0.291728309 |
|  | 1105 | 2.76287E-08 | 0.243853739 |
|  | 1106 | 1.85866E-08 | 0.241225291 |
|  | 1107 | 0.000751514 | 0.063164362 |
|  | 1108 | 0.00012867 | 0.059329133 |
|  | 1109 | 0.000694269 | 0.079763308 |
|  | 1110 | 9.07208E-05 | 0.14743394 |
|  | 1111 | 0.000209036 | 0.122576257 |
|  | 1112 | 1.7509E-06 | 0.097777962 |
|  | 1113 | 0.025824169 | 0.009856683 |
|  | 1114 | 8.62366E-06 | 0.030250691 |
|  | 1115 | 0.000102276 | 0.034073029 |
|  | 1116 | 3.00851E-06 | 0.038690054 |
|  | 1117 | 0.001748382 | 0.014727146 |
|  | 1118 | 0.002770416 | 0.005717083 |
|  | 1119 | 0.01480438 | 0.004467758 |
|  | 1120 | 0.042800028 | 0.000584485 |
|  | 1121 | 7.51769E-05 | 0.010311085 |
|  | 1122 | 0.006314774 | 0.004460465 |
| TUEN MUN | 1123 | 0.219392355 | 0.001442501 |
|  | 1124 | 0.017216959 | 0.009216449 |
|  | 1125 | 4.95658E-06 | 0.091593475 |
|  | 1126 | 2.10028E-06 | 0.094925653 |
|  | 1127 | 5.91228E-06 | 0.075916958 |
|  | 1128 | 3.62815E-05 | 0.048143003 |
|  | 1129 | 0.000104575 | 0.060262569 |
|  | 1130 | 4.61607E-05 | 0.080851581 |
|  | 1131 | 3.84239E-06 | 0.058645785 |
|  | 1132 | 3.44454E-06 | 0.093418171 |
|  | 1133 | 6.00499E-05 | 0.062152653 |
|  | 1134 | 0.010446365 | 0.091008046 |
|  | 1135 | 0.000190504 | 0.147914796 |
|  | 1136 | 1.72312E-05 | 0.152282114 |
|  | 1137 | 2.52225E-05 | 0.175912378 |
|  | 1138 | 1.93441E-05 | 0.110592008 |
|  | 1139 | 8.24314E-06 | 0.139238761 |
|  | 1140 | 7.51123E-06 | 0.108771462 |
|  | 1141 | 5.00493E-05 | 0.139967348 |
|  | 1142 | 3.30143E-05 | 0.066570014 |
|  | 1143 | 4.54984E-05 | 0.064370497 |
|  | 1144 | 1.38892E-05 | 0.049675703 |
|  | 1145 | 0.001460638 | 0.065210572 |
|  | 1146 | 7.92636E-05 | 0.129393734 |
|  | 1147 | 3.14977E-05 | 0.097248497 |
|  | 1148 | 1.85464E-06 | 0.119756786 |
|  | 1149 | 0.00028084 | 0.054971844 |
|  | 1150 | 0.002500415 | 0.031840877 |
|  | 1151 | 7.3977E-05 | 0.141184952 |
|  | 1152 | 6.24594E-05 | 0.173813298 |
|  | 1153 | 7.03534E-05 | 0.183143575 |
|  | 1154 | 0.000169077 | 0.179251795 |
|  | 1155 | 5.72481E-05 | 0.19319091 |
|  | 1156 | 0.000170252 | 0.193206572 |
|  | 1157 | 0.000607379 | 0.165181383 |
|  | 1158 | 0.000438598 | 0.151526964 |
|  | 1159 | 5.59425E-05 | 0.11844627 |
|  | 1160 | 0.000234118 | 0.208659009 |
|  | 1161 | 1.44508E-05 | 0.205651809 |
|  | 1162 | 6.07477E-05 | 0.152394303 |
|  | 1163 | 6.79226E-05 | 0.098115298 |
|  | 1164 | 8.88046E-05 | 0.158911314 |
|  | 1165 | 0.000633603 | 0.185780263 |
|  | 1166 | 0.000650607 | 0.047157922 |
|  | 1167 | 0.000845063 | 0.111134978 |
|  | 1168 | 0.001199249 | 0.076216096 |
|  | 1169 | 0.001430556 | 0.111134138 |
|  | 1170 | 0.000440518 | 0.085134654 |
|  | 1171 | 4.24753E-05 | 0.081838741 |
|  | 1172 | 0.00076851 | 0.058811312 |
|  | 1173 | 1.19599E-05 | 0.074733113 |
|  | 1174 | 2.64331E-06 | 0.111898425 |
|  | 1175 | 0.000233426 | 0.030201521 |
|  | 1176 | 0.000468035 | 0.033451502 |
|  | 1177 | 0.000189874 | 0.027379415 |
|  | 1178 | 0.000325654 | 0.034502126 |
|  | 1179 | 0.00017927 | 0.049294415 |
|  | 1180 | 0.000728234 | 0.057188313 |
|  | 1181 | 0.001002555 | 0.095851516 |
|  | 1182 | 4.3799E-05 | 0.04493819 |
|  | 1183 | 0.001875479 | 0.015762791 |
|  | 1184 | 5.46272E-05 | 0.011066851 |
|  | 1185 | 0.000731236 | 0.011543603 |
|  | 1186 | 0.003788089 | 0.004824634 |
|  | 1187 | 5.77075E-05 | 0.006560863 |
|  | 1188 | 0.000502847 | 0.005131332 |
|  | 1189 | 0.000497592 | 0.00311057 |
|  | 1190 | 0.001477027 | 0.001877991 |
|  | 1191 | 0 | 0.012849529 |
|  | 1192 | 0.139314053 | 0.001838071 |
|  | 1193 | 0.000626246 | 0.001769331 |
|  | 1194 | 1.17728E-05 | 0.002776188 |
|  | 1195 | 0.273622068 | 9.77859E-05 |
|  | 1196 | 2.08446E-06 | 0.010044415 |
|  | 1197 | 0.005682125 | 0.014311779 |
|  | 1198 | 0.005851468 | 0.014947244 |
|  | 1199 | 0.000413922 | 0.015431812 |
|  | 1200 | 0 | 0.017326546 |
|  | 1201 | 0.000946414 | 0.016270614 |
|  | 1202 | 0.004688304 | 0.004869502 |
|  | 1203 | 0.002385562 | 0.014549831 |
| WAN CHAI | 1204 | 4.24477E-08 | 0.247807884 |
|  | 1205 | 2.64959E-06 | 0.52869377 |
|  | 1206 | 1.09849E-05 | 0.572157509 |
|  | 1207 | 8.53977E-09 | 0.210785125 |
|  | 1208 | 0 | 0.493822234 |
|  | 1209 | 2.08471E-08 | 0.209061106 |
|  | 1210 | 2.12992E-07 | 0.473154782 |
|  | 1211 | 1.70645E-06 | 0 |
|  | 1212 | 0 | 0.343288033 |
|  | 1213 | 7.53509E-09 | 0.367104965 |
|  | 1214 | 8.53977E-09 | 0.130167792 |
|  | 1215 | 0 | 0.354409282 |
|  | 1216 | 1.16091E-06 | 0 |
|  | 1217 | 2.01438E-07 | 0.233885098 |
|  | 1218 | 1.06546E-05 | 0.174330585 |
|  | 1219 | 0 | 0.167654451 |
|  | 1220 | 9.8137E-06 | 0.170874602 |
|  | 1221 | 0.000113612 | 0.164837701 |
|  | 1222 | 0.000207792 | 0.113321518 |
|  | 1223 | 2.53933E-06 | 0.272373446 |
|  | 1224 | 8.29111E-06 | 0.597168017 |
|  | 1225 | 7.71091E-06 | 0.311590959 |
|  | 1226 | 4.84732E-06 | 0.35400951 |
|  | 1227 | 1.1702E-05 | 0.187569032 |
|  | 1228 | 4.47283E-06 | 0.243123153 |
|  | 1229 | 2.56258E-05 | 0.312642297 |
|  | 1230 | 0.000104564 | 0.403564089 |
|  | 1231 | 8.37073E-06 | 0 |
|  | 1232 | 1.88754E-05 | 0.573742978 |
|  | 1233 | 1.65137E-05 | 0.600477613 |
|  | 1234 | 1.13815E-05 | 0.306069979 |
|  | 1235 | 6.72055E-06 | 0 |
|  | 1236 | 0 | 0.614971526 |
|  | 1237 | 1.19258E-05 | 0.612714915 |
|  | 1238 | 0 | 0.32023199 |
|  | 1239 | 4.41019E-05 | 0.445221042 |
|  | 1240 | 0.0026491 | 0.129855044 |
|  | 1241 | 0.000283988 | 0.222182958 |
|  | 1242 | 3.07826E-05 | 0.460940258 |
|  | 1243 | 0.000426536 | 0.218169857 |
|  | 1244 | 5.0723E-05 | 0.306740917 |
|  | 1245 | 0.000190085 | 0.212141367 |
|  | 1246 | 0.000189911 | 0.139757688 |
|  | 1247 | 0.000130832 | 0.176512137 |
|  | 1248 | 0.000555691 | 0.061745784 |
|  | 1249 | 6.35186E-05 | 0.064752482 |
|  | 1250 | 7.14623E-05 | 0.064109188 |
|  | 1251 | 8.82515E-05 | 0.061062366 |
|  | 1252 | 9.40558E-05 | 0.061736905 |
|  | 1253 | 0.000131679 | 0.057657004 |
|  | 1254 | 0.000174999 | 0.070843191 |
|  | 1255 | 6.54445E-05 | 0 |
|  | 1256 | 0.000125923 | 0.060803336 |
|  | 1257 | 0.000787148 | 0.056006641 |
|  | 1258 | 0.000114755 | 0.056425411 |
|  | 1259 | 0.001116107 | 0.065653984 |
|  | 1260 | 8.90698E-06 | 0.017724867 |
|  | 1261 | 6.91546E-06 | 0.110105773 |
|  | 1262 | 0.000462983 | 0.051268447 |
|  | 1263 | 9.08506E-05 | 0.085893947 |
|  | 1264 | 0.000465629 | 0.062576136 |
|  | 1265 | 7.73653E-06 | 0.50982269 |
|  | 1266 | 4.32364E-06 | 0.23683273 |
|  | 1267 | 2.35974E-06 | 0.272246785 |
|  | 1268 | 7.59261E-06 | 0.133536519 |
|  | 1269 | 4.99351E-06 | 0.458316321 |
|  | 1270 | 3.7359E-06 | 0.294954219 |
|  | 1271 | 1.04467E-05 | 0.255618941 |
|  | 1272 | 2.63944E-05 | 0 |
|  | 1273 | 0.000240556 | 0.179526721 |
|  | 1274 | 2.37154E-06 | 0.291832905 |
|  | 1275 | 1.02796E-05 | 0.08244599 |
|  | 1276 | 0.000665462 | 0.030725664 |
|  | 1277 | 0.000436324 | 0.030276834 |
|  | 1278 | 0.0010834 | 0.025985359 |
|  | 1279 | 0.023005472 | 0.034769115 |
|  | 1280 | 0.001173788 | 0.072895733 |
|  | 1281 | 0.018197887 | 0.017681902 |
| WONG TAI SIN | 1282 | 0.004790189 | 0.272179734 |
|  | 1283 | 4.69838E-06 | 0.518097243 |
|  | 1284 | 1.02485E-05 | 0.512341578 |
|  | 1285 | 3.25014E-06 | 0.652280525 |
|  | 1286 | 4.20986E-06 | 0.682868499 |
|  | 1287 | 2.51295E-06 | 0.624710709 |
|  | 1288 | 3.54752E-06 | 0.49651608 |
|  | 1289 | 2.57198E-07 | 0.593545059 |
|  | 1290 | 1.62004E-07 | 0.295930386 |
|  | 1291 | 1.75844E-06 | 0.473971683 |
|  | 1292 | 9.64241E-07 | 0 |
|  | 1293 | 7.12317E-07 | 0.428483858 |
|  | 1294 | 6.59898E-06 | 0.351975272 |
|  | 1295 | 0 | 0.303637362 |
|  | 1296 | 0.000279939 | 0.32909145 |
|  | 1297 | 6.27924E-08 | 0.575327218 |
|  | 1298 | 0 | 0.42665614 |
|  | 1299 | 5.1585E-05 | 0.325589041 |
|  | 1300 | 4.6484E-06 | 0.272841203 |
|  | 1301 | 7.35099E-06 | 0.154382073 |
|  | 1302 | 3.82105E-06 | 0.313147402 |
|  | 1303 | 2.42856E-06 | 0.61598196 |
|  | 1304 | 8.75075E-07 | 0.685160111 |
|  | 1305 | 5.08619E-06 | 0.665472198 |
|  | 1306 | 2.72343E-06 | 0.182727427 |
|  | 1307 | 5.2183E-06 | 0.204424425 |
|  | 1308 | 6.50605E-06 | 0.120994224 |
|  | 1309 | 0.000960547 | 0.063585273 |
|  | 1310 | 2.00634E-05 | 0.142272735 |
|  | 1311 | 1.66548E-05 | 0.155703765 |
|  | 1312 | 3.3179E-05 | 0.079490421 |
|  | 1313 | 2.45644E-05 | 0.079522128 |
|  | 1314 | 6.91696E-06 | 0.125095116 |
|  | 1315 | 1.13579E-06 | 0.14936003 |
|  | 1316 | 1.2247E-06 | 0.27810352 |
|  | 1317 | 5.53201E-06 | 0.359280053 |
|  | 1318 | 1.7663E-05 | 0.29525366 |
|  | 1319 | 4.71697E-07 | 0.302311768 |
|  | 1320 | 2.87539E-06 | 0.337355063 |
|  | 1321 | 2.24445E-06 | 0.314131162 |
|  | 1322 | 5.00104E-06 | 0.360887023 |
|  | 1323 | 5.10653E-06 | 0.364269206 |
|  | 1324 | 4.64011E-06 | 0.330552014 |
|  | 1325 | 1.39279E-05 | 0.188174649 |
|  | 1326 | 1.62745E-05 | 0.239789376 |
|  | 1327 | 2.49555E-05 | 0.308137238 |
|  | 1328 | 8.73568E-07 | 0.333238572 |
|  | 1329 | 2.70183E-06 | 0.242414892 |
|  | 1330 | 3.62865E-06 | 0.118834356 |
|  | 1331 | 2.84299E-06 | 0.357313835 |
|  | 1332 | 1.84208E-06 | 0.174827033 |
|  | 1333 | 1.16093E-05 | 0.349568622 |
|  | 1334 | 1.17168E-05 | 0.344371981 |
|  | 1335 | 2.05937E-05 | 0.168990492 |
|  | 1336 | 1.40399E-05 | 0.326575431 |
|  | 1337 | 1.54628E-05 | 0.413003437 |
|  | 1338 | 0.0090918 | 0.090956903 |
|  | 1339 | 8.85579E-05 | 0.165666468 |
|  | 1340 | 1.47605E-05 | 0.208681437 |
|  | 1341 | 5.51345E-05 | 0.271174335 |
|  | 1342 | 0.000138652 | 0.351526912 |
|  | 1343 | 7.18526E-05 | 0.432643043 |
|  | 1344 | 0.000897 | 0.252656084 |
|  | 1345 | 0.000155057 | 0.13541977 |
|  | 1346 | 8.34823E-05 | 0.112464503 |
|  | 1347 | 6.93914E-05 | 0.117465154 |
|  | 1348 | 0 | 0.109511314 |
|  | 1349 | 8.41042E-05 | 0 |
| YAU TSIM MONG | 1350 | 0 | 0.24742325 |
|  | 1351 | 0 | 0.770921906 |
|  | 1352 | 0 | 0.389769411 |
|  | 1353 | 1.50451E-07 | 0.647025757 |
|  | 1354 | 0 | 0.242435977 |
|  | 1355 | 0 | 0.559611336 |
|  | 1356 | 0 | 0.447565431 |
|  | 1357 | 0 | 0.331683703 |
|  | 1358 | 0 | 0.370638902 |
|  | 1359 | 1.61377E-06 | 0.605611115 |
|  | 1360 | 0 | 0.717730764 |
|  | 1361 | 0 | 0.374944341 |
|  | 1362 | 0 | 0.520359798 |
|  | 1363 | 0 | 0.445887872 |
|  | 1364 | 0 | 0.626241524 |
|  | 1365 | 0 | 0.610648684 |
|  | 1366 | 0 | 0.568038832 |
|  | 1367 | 0 | 0.439214809 |
|  | 1368 | 0 | 0.445726155 |
|  | 1369 | 0 | 0.321973543 |
|  | 1370 | 0 | 0.248942335 |
|  | 1371 | 0 | 0.503154251 |
|  | 1372 | 5.60109E-08 | 0.56536846 |
|  | 1373 | 1.33873E-07 | 0.303050964 |
|  | 1374 | 9.20286E-07 | 0 |
|  | 1375 | 7.37836E-06 | 0.29740269 |
|  | 1376 | 1.92695E-05 | 0.281514141 |
|  | 1377 | 1.63562E-06 | 0.433950249 |
|  | 1378 | 1.56228E-07 | 0.379709588 |
|  | 1379 | 6.17878E-07 | 0.620995106 |
|  | 1380 | 4.99702E-06 | 0.616581608 |
|  | 1381 | 2.83319E-07 | 0.304737662 |
|  | 1382 | 2.39365E-07 | 0.199985718 |
|  | 1383 | 1.60497E-07 | 0.603094193 |
|  | 1384 | 5.64378E-07 | 0.619550796 |
|  | 1385 | 1.60422E-06 | 0.357748555 |
|  | 1386 | 3.07181E-07 | 0.323128617 |
|  | 1387 | 5.98286E-06 | 0 |
|  | 1388 | 6.11046E-06 | 0.176914377 |
|  | 1389 | 3.43701E-06 | 0.412120205 |
|  | 1390 | 4.12622E-06 | 0 |
|  | 1391 | 6.68112E-08 | 0.454623472 |
|  | 1392 | 4.81995E-07 | 0.468983774 |
|  | 1393 | 9.20437E-06 | 0 |
|  | 1394 | 6.85467E-06 | 0.343360524 |
|  | 1395 | 2.53933E-06 | 0.239419238 |
|  | 1396 | 4.54366E-07 | 0.303877306 |
|  | 1397 | 6.88456E-07 | 0 |
|  | 1398 | 1.58237E-07 | 0.671688305 |
|  | 1399 | 5.04349E-07 | 0.6042578 |
|  | 1400 | 1.3376E-05 | 0 |
|  | 1401 | 9.92372E-07 | 0.693466238 |
|  | 1402 | 6.89963E-07 | 0.558489579 |
|  | 1403 | 2.21406E-06 | 0.557453235 |
|  | 1404 | 0 | 0.462138952 |
|  | 1405 | 0 | 0.701913191 |
|  | 1406 | 0 | 0.853413265 |
|  | 1407 | 1.18828E-05 | 0.202554663 |
|  | 1408 | 7.00512E-07 | 0.484844615 |
|  | 1409 | 8.37475E-06 | 0.312928065 |
|  | 1410 | 1.51267E-05 | 0.146361379 |
|  | 1411 | 2.42655E-05 | 0.261678807 |
|  | 1412 | 9.18729E-06 | 0.457328313 |
|  | 1413 | 7.49918E-06 | 0.379393626 |
|  | 1414 | 7.58784E-07 | 0.60603264 |
|  | 1415 | 4.51704E-06 | 0.469900319 |
|  | 1416 | 7.36681E-07 | 0 |
|  | 1417 | 1.99836E-05 | 0.666366671 |
|  | 1418 | 6.53092E-06 | 0.409564387 |
|  | 1419 | 1.36915E-05 | 0.842577465 |
|  | 1420 | 1.8305E-05 | 0.864102343 |
|  | 1421 | 2.50326E-05 | 0 |
|  | 1422 | 1.04811E-05 | 0.462778266 |
|  | 1423 | 9.22125E-05 | 0.255259426 |
|  | 1424 | 1.396E-06 | 0.425439739 |
|  | 1425 | 0 | 0.310687734 |
|  | 1426 | 6.58065E-07 | 0.45102163 |
|  | 1427 | 2.46046E-06 | 0 |
|  | 1428 | 2.62171E-06 | 0 |
|  | 1429 | 1.58413E-06 | 0.383913274 |
|  | 1430 | 5.57823E-06 | 0 |
|  | 1431 | 4.08477E-06 | 0 |
|  | 1432 | 5.51443E-06 | 0.288227393 |
|  | 1433 | 3.54325E-06 | 0.601038971 |
|  | 1434 | 4.38969E-06 | 0.510098614 |
|  | 1435 | 2.76312E-06 | 0 |
|  | 1436 | 2.64331E-06 | 0 |
|  | 1437 | 1.71273E-06 | 0.669081404 |
|  | 1438 | 1.28122E-06 | 0 |
|  | 1439 | 7.34923E-07 | 0.183165282 |
|  | 1440 | 6.79414E-07 | 0.512432715 |
|  | 1441 | 5.98286E-07 | 0.297502842 |
|  | 1442 | 0 | 0.386945854 |
|  | 1443 | 3.14967E-07 | 0.365389093 |
|  | 1444 | 2.11761E-06 | 0.72801935 |
|  | 1445 | 1.85112E-07 | 0 |
|  | 1446 | 0.000131992 | 0.644320889 |
|  | 1447 | 0.000191669 | 0.284760592 |
|  | 1448 | 4.79267E-05 | 0.483870386 |
|  | 1449 | 5.24468E-06 | 0.386402147 |
|  | 1450 | 0 | 0.397069467 |
|  | 1451 | 5.87486E-07 | 0.678126602 |
|  | 1452 | 9.06271E-06 | 0.667877506 |
|  | 1453 | 1.82475E-06 | 0.492411597 |
|  | 1454 | 1.79385E-06 | 0.527972467 |
|  | 1455 | 3.23255E-07 | 1 |
|  | 1456 | 1.16643E-05 | 0.931157842 |
|  | 1457 | 0 | 0.839932768 |
|  | 1458 | 1.63582E-05 | 0 |
|  | 1459 | 0 | 0 |
|  | 1460 | 4.90359E-06 | 0.780114529 |
|  | 1461 | 1.9109E-06 | 0.41181856 |
|  | 1462 | 1.23651E-06 | 0.64855418 |
|  | 1463 | 2.65285E-06 | 0.722697441 |
|  | 1464 | 2.58454E-07 | 0.81121371 |
|  | 1465 | 0 | 0.2876959 |
|  | 1466 | 0 | 0.577328625 |
|  | 1467 | 0 | 0.504032489 |
|  | 1468 | 0 | 0.845171688 |
|  | 1469 | 0 | 0.856443186 |
|  | 1470 | 0 | 0.388093459 |
|  | 1471 | 2.28414E-06 | 0.853188633 |
|  | 1472 | 0 | 0.457771407 |
|  | 1473 | 6.37034E-05 | 0.090226862 |
|  | 1474 | 2.73675E-06 | 0.367137204 |
|  | 1475 | 8.97455E-06 | 0.440850317 |
|  | 1476 | 2.9356E-05 | 0.22139533 |
|  | 1477 | 5.82086E-06 | 0.603331855 |
|  | 1478 | 5.5442E-05 | 0.27254499 |
|  | 1479 | 2.77216E-05 | 0.49469528 |
|  | 1480 | 9.39804E-05 | 0.077787483 |
|  | 1481 | 9.53842E-06 | 0.050076025 |
|  | 1482 | 1.72689E-05 | 0.123389673 |
|  | 1483 | 5.60058E-06 | 0.339184115 |
|  | 1484 | 1.0635E-05 | 0.222300179 |
| YUEN LONG | 1485 | 1.41431E-05 | 0.055989942 |
|  | 1486 | 6.71553E-05 | 0.061103661 |
|  | 1487 | 4.70393E-05 | 0.058821212 |
|  | 1488 | 6.59363E-05 | 0.062192557 |
|  | 1489 | 3.30087E-06 | 0.072208355 |
|  | 1490 | 4.52432E-06 | 0.089617212 |
|  | 1491 | 2.51554E-05 | 0.08035295 |
|  | 1492 | 1.5894E-06 | 0.098959595 |
|  | 1493 | 6.19334E-06 | 0.115571297 |
|  | 1494 | 2.81468E-05 | 0.050187641 |
|  | 1495 | 3.07238E-05 | 0.051658942 |
|  | 1496 | 2.83319E-07 | 0.10054569 |
|  | 1497 | 2.72795E-05 | 0.064148422 |
|  | 1498 | 6.68109E-05 | 0.085519143 |
|  | 1499 | 2.49402E-05 | 0.131348123 |
|  | 1500 | 6.65725E-06 | 0.119471763 |
|  | 1501 | 1.36636E-05 | 0.083712669 |
|  | 1502 | 0.000103146 | 0.078880961 |
|  | 1503 | 2.71585E-05 | 0.120539253 |
|  | 1504 | 3.69338E-05 | 0.074470924 |
|  | 1505 | 4.58214E-05 | 0.090552433 |
|  | 1506 | 6.74592E-05 | 0.047536558 |
|  | 1507 | 3.67155E-05 | 0.004506446 |
|  | 1508 | 0.000168001 | 0.009802936 |
|  | 1509 | 0.002056537 | 0.008350138 |
|  | 1510 | 0.002401943 | 0.001837157 |
|  | 1511 | 0.001085159 | 0.019620415 |
|  | 1512 | 0.001175862 | 0.003361695 |
|  | 1513 | 0.00786369 | 0.007152663 |
|  | 1514 | 3.27184E-05 | 0.031550937 |
|  | 1515 | 0.073711452 | 0.001636018 |
|  | 1516 | 0.012680186 | 0.007109419 |
|  | 1517 | 2.21986E-05 | 0.031521699 |
|  | 1518 | 0.001365939 | 0.027283122 |
|  | 1519 | 0.00086248 | 0.032126206 |
|  | 1520 | 0.000367478 | 0.022088131 |
|  | 1521 | 0.000451637 | 0.016385596 |
|  | 1522 | 0.000212924 | 0.022027292 |
|  | 1523 | 0.000176876 | 0.037049544 |
|  | 1524 | 0.010184176 | 0.001823125 |
|  | 1525 | 0.000307062 | 0.003590415 |
|  | 1526 | 0.017142032 | 0.016724556 |
|  | 1527 | 0.000892451 | 0.006299988 |
|  | 1528 | 0.013402522 | 0.005858216 |
|  | 1529 | 0.000739121 | 0.016045759 |
|  | 1530 | 0.000157399 | 0.022967952 |
|  | 1531 | 3.01979E-05 | 0.028161788 |
|  | 1532 | 0.000246663 | 0.027018076 |
|  | 1533 | 0.002252372 | 0.00495289 |
|  | 1534 | 0.001883727 | 0.013715164 |
|  | 1535 | 0.00027936 | 0.014970215 |
|  | 1536 | 0.000671622 | 0.015817059 |
|  | 1537 | 0.000766596 | 0.011232218 |
|  | 1538 | 0.000253173 | 0.013832882 |
|  | 1539 | 0.098348686 | 0.001195396 |
|  | 1540 | 0.013821366 | 0.003097521 |
|  | 1541 | 0.000399067 | 0.006333546 |
|  | 1542 | 0.000121067 | 0.00930376 |
|  | 1543 | 0.00017628 | 0.007081968 |
|  | 1544 | 8.27416E-05 | 0.00990302 |
|  | 1545 | 0.000211834 | 0.011217295 |
|  | 1546 | 0.005005324 | 0.006997284 |
|  | 1547 | 0.000341119 | 0.01139443 |
|  | 1548 | 0.000319009 | 0.013081286 |
|  | 1549 | 0.00078558 | 0.009465369 |
|  | 1550 | 0.011079563 | 0.012811502 |
|  | 1551 | 2.57899E-05 | 0.214445861 |
|  | 1552 | 0.000140685 | 0.142470265 |
|  | 1553 | 5.60209E-06 | 0.214380078 |
|  | 1554 | 3.58696E-06 | 0.229443351 |
|  | 1555 | 9.99379E-06 | 0.23009439 |
|  | 1556 | 7.31708E-06 | 0.224007469 |
|  | 1557 | 8.49707E-07 | 0.225770101 |
|  | 1558 | 3.37773E-06 | 0.216115558 |
|  | 1559 | 1.26916E-06 | 0.213993812 |
|  | 1560 | 2.17588E-06 | 0.209474822 |
|  | 1561 | 3.4669E-06 | 0.180576359 |
|  | 1562 | 8.88764E-06 | 0.193331644 |
|  | 1563 | 7.28518E-06 | 0.18682895 |
|  | 1564 | 0.000121209 | 0.145152308 |
|  | 1565 | 4.30819E-05 | 0.184514623 |
|  | 1566 | 1.9428E-05 | 0.205546408 |
|  | 1567 | 0.000145828 | 0.080293721 |
|  | 1568 | 2.97835E-05 | 0.163629758 |
|  | 1569 | 0.000267134 | 0.123183381 |
|  | 1570 | 0.000792739 | 0.04196514 |
|  | 1571 | 4.25158E-05 | 0.089572621 |
|  | 1572 | 1.36611E-06 | 0.196111251 |
|  | 1573 | 3.24742E-05 | 0.157634383 |
|  | 1574 | 0.01648736 | 0.009315824 |
|  | 1575 | 0.057598472 | 0.002755809 |
|  | 1576 | 9.77628E-06 | 0.074818964 |
|  | 1577 | 8.57722E-05 | 0.12202628 |
|  | 1578 | 1.54484E-05 | 0.196794659 |
|  | 1579 | 1.73061E-05 | 0.193207291 |
|  | 1580 | 2.13439E-05 | 0.091147794 |
|  | 1581 | 1.72878E-05 | 0.035984064 |
|  | 1582 | 0.000456887 | 0.140985975 |
|  | 1583 | 2.24036E-05 | 0.108690974 |
|  | 1584 | 2.15986E-05 | 0.179857312 |
|  | 1585 | 2.33317E-05 | 0.164147492 |
|  | 1586 | 0.001871024 | 0.078692497 |
|  | 1587 | 0.000126739 | 0.023790823 |
|  | 1588 | 0.000102664 | 0.094418119 |
|  | 1589 | 3.40913E-05 | 0.060628562 |
|  | 1590 | 0.000493925 | 0.006005825 |
|  | 1591 | 6.24388E-05 | 0.086279216 |
|  | 1592 | 0.000327349 | 0.09111337 |
|  | 1593 | 4.01698E-05 | 0.108423137 |
|  | 1594 | 3.14186E-05 | 0 |
|  | 1595 | 0.000622519 | 0.065000534 |
|  | 1596 | 0.087777007 | 0.002735276 |
|  | 1597 | 0.000638407 | 0.007162013 |
|  | 1598 | 0.000193296 | 0.00208681 |
|  | 1599 | 0.002103943 | 0.00980608 |
|  | 1600 | 0.000203367 | 0.001500594 |
|  | 1601 | 0.002103943 | 0.002761055 |
|  | 1602 | 0.000301693 | 0.013037499 |
|  | 1603 | 0.016163647 | 0.001238518 |
|  | 1604 | 0.00220444 | 0.001822674 |
|  | 1605 | 0.016160508 | 0.00421587 |
|  | 1606 | 0.014297132 | 0.004907982 |
|  | 1607 | 0.059697936 | 0.001767034 |
|  | 1608 | 0.000330272 | 0.000518673 |
|  | 1609 | 0.000467442 | 0.002198513 |
|  | 1610 | 0.000421641 | 0.001523248 |
|  | 1611 | 0.141723182 | 0.000596446 |
|  | 1612 | 0.001442557 | 0.004824587 |
|  | 1613 | 0.009906009 | 0.004483174 |
|  | 1614 | 0.000140412 | 0.004389078 |
|  | 1615 | 0.006086537 | 0.004093518 |
|  | 1616 | 0.022672714 | 0.000796467 |
|  | 1617 | 0.011288492 | 0.001436266 |
|  | 1618 | 0.003941233 | 0.002866024 |
|  | 1619 | 0 | 0.007075449 |
|  | 1620 | 0.046170895 | 0.001442723 |
|  | 1621 | 0.000564946 | 0.004076966 |
|  | 1622 | 0.020286642 | 0.002999492 |
